# Optimization of Xylooligosaccharides Production by Native and Recombinant Xylanase Hydrolysis of Chicken Feed Substrates

**DOI:** 10.3390/ijms242317110

**Published:** 2023-12-04

**Authors:** Priyashini Dhaver, Brett Pletschke, Bruce Sithole, Roshini Govinden

**Affiliations:** 1 Discipline of Microbiology, School of Life Sciences, Westville Campus, University of KwaZulu-Natal, Durban 4000, South Africa; pdhaver10@gmail.com; 2 Enzyme Science Programme (ESP), Department of Biochemistry and Microbiology, Rhodes University, Makhanda (Grahamstown) 6140, South Africa; b.pletschke@ru.ac.za; 3 Biorefinery Industry Development Facility, Council for Scientific and Industrial Research, Durban 4000, South Africa; bbsithole@hotmail.com; 4 Discipline of Chemical Engineering, University of KwaZulu-Natal, Durban 4000, South Africa

**Keywords:** xylooligosaccharides, chicken feed, lignocellulosic biomass, response surface methodology, xylanase

## Abstract

Poultry production faces several challenges, with feed efficiency being the main factor that can be influenced through the use of different nutritional strategies. Xylooligosaccharides (XOS) are functional feed additives that are attracting growing commercial interest due to their excellent ability to modulate the composition of the gut microbiota. The aim of the study was to apply crude and purified fungal xylanases, from *Trichoderma harzianum,* as well as a recombinant glycoside hydrolase family 10 xylanase, derived from *Geobacillus stearothermophilus* T6, as additives to locally produced chicken feeds. A Box–Behnken Design (BBD) was used to optimize the reducing sugar yield. Response surface methodology (RSM) revealed that reducing sugars were higher (8.05 mg/mL, 2.81 mg/mL and 2.98 mg/mL) for the starter feed treated with each of the three enzymes compared to the treatment with grower feed (3.11 mg/mL, 2.41 mg/mL and 2.62 mg/mL). The hydrolysis products were analysed by thin-layer chromatography (TLC), and high-performance liquid chromatography (HPLC) analysis and showed that the enzymes hydrolysed the chicken feeds, producing a range of monosaccharides (arabinose, mannose, glucose, and galactose) and XOS, with xylobiose being the predominant XOS. These results show promising data for future applications as additives to poultry feeds.

## 1. Introduction

Xylan is the second most common renewable terrestrial polysaccharide in nature after cellulose. Non-starch polysaccharides (NSPs) like xylan cannot be hydrolysed by endogenous enzymes in monogastric animals like poultry [1]. This leads to an environment favourable for these NSPs to encapsulate the other nutrients, thus acting as a barrier in the small intestine and resulting in the increased viscosity of the digesta [2]. Recent studies [3,4] have shown that a decreased growth performance due to increased digesta viscosity has been commonly seen in chickens that ingest diets containing high levels of NSPs.

In response to this, livestock producers have incorporated exogenous enzymes such as xylanases into poultry feeds in order to degrade the xylan into short-chain sugars, thereby reducing intestinal viscosity and enhancing the digestive utilization of nutrients [5]. Research suggests that the presence of certain enzymes, such as xylanase, or the combination of enzymes with dietary components, like xylanase with hybrid rye, can have an impact on the integrity of the chicken intestinal barrier, specifically affecting the tight junction proteins [6].

Xylan, derived from lignocellulosic biomass, can be hydrolysed through the use of exogenous chemicals and enzymatic processes to generate a mixture of xylooligosaccharides (XOSs) and monosaccharides [7,8]. The resulting XOS mixture is recognized as a prebiotic [1]. XOS are oligosaccharides made up of repeating xylose units linked by β-(1–4)-linkages—examples include xylobiose, xylotriose, xylotetraose, xylopentaose, and xylohexaose [9]. XOS have a promising market potential as food additives, feed additives, health care products, and pharmaceuticals [10] because of their prebiotic [11], antioxidant [12], and anticancer activity [13]. Hemicelluloses are efficiently hydrolysed into monosaccharides or XOS with minimal enzyme loading which is important for the lignocellulosic bioenergy and biorefinery industry [8].

Lignocellulosic biomass is the most cost-efficient and sustainable natural resource available globally. It is comprised of terrestrial vegetation like shrubs and grasses, as well as agricultural biomass by-products like corn stover, straw, saw-dust wastes, paper mill discards, and energy-yielding vegetation [14]. Its hydrolysis products have been widely adopted as prebiotics and carbohydrases in feed additives in broiler chickens, to enhance intestinal health and stimulate performance [15]. However, hydrolysis conditions affect the production of hydrolysis products. Therefore, it is vital to understand the effects of enzyme dosage, feed substrate loading, incubation time, temperature, and pH during hydrolysis [16,17]. An efficient way to understand the impact of various process variables and their interactions on the process’s outcome and to identify the ideal conditions for maximizing the process output is to use the Response Surface Methodology (RSM), a mathematical and statistical technique [18]. The RSM, using a Box Behnken Design (BBD), is an effective optimization tool. The RSM design can provide the dependence of enzyme activity on independent variables (enzyme dosage, feed loading, incubation time, pH, and incubation temperature), predicted results for responses, and levels for independent variables in the form of mathematical models [19].

Following optimization to enhance yields, analysis of the hydrolysates is usually carried out using two techniques: thin-layer chromatography (TLC) and high-performance liquid chromatography (HPLC) [20,21,22]. HPLC employs detectors [such as refractive index (RI) and diode array detector (DAD)] for the determination of total component sugars produced after hydrolysis [23,24]. 

The addition of xylanases to chicken cereal feed diets can enhance NSP hydrolysis and the production of prebiotic XOSs. Considering the potential market demand for XOSs in the agricultural and pharmaceutical industry, the aim of the present study was to optimize the hydrolysis of starter and grower chicken feed using crude [25] and purified [19] *Trichoderma harzianum* xylanases and the recombinant *Geobacillus stearothermophilus* XT6 xylanase [26] obtained from previous studies, to enhance the production of reducing sugars. XOSs and monosaccharides were monitored qualitatively and quantitatively using chromatographic techniques to analyse the feed hydrolysate profiles. 

## 2. Results and Discussion

### 2.1. Optimization of Feed Hydrolysis for Enhanced Reducing Sugars

#### 2.1.1. Hydrolysis of Starter and Grower Feeds with Crude *T. harzianum* Xylanase

This study focused on the conditions favouring the hydrolysis of chicken feeds. Production of XOS from various sources of xylan such as wheat bran, birchwood, corncob, tobacco stalk, etc., using commercial xylanases has been reported previously [27,28]. However, relatively few studies have involved the xylanases from *T. harzianum* and the recombinant XT6 xylanase. The runs that produced the highest reducing sugars for the starter feed with the crude *T. harzianum* xylanase was run 45 (8.05 mg/mL) with all variables at their optimal levels (Table 1). 

**Figure 1:**
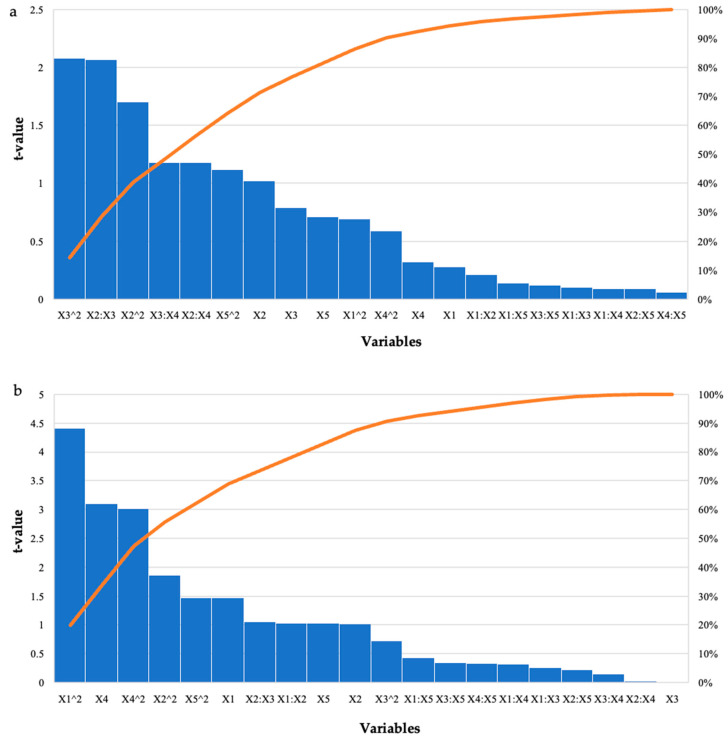
Pareto chart of standardized effects for the BBD for enzyme dosage (X1), feed loading (X2), incubation time (X3), incubation temperature (X4), and pH (X5) for the hydrolysis of (**a**) starter feed and (**b**) grower feed with crude *T. harzianum* xylanase. The orange line represents *p* = 0.05.

For the grower feed, the highest reducing sugars was produced in run 1 (3.21 mg/mL) with the enzyme dosage (5 U/mL) and feed percentage (0.5%) at their low levels and incubation time, pH, and incubation temperature at their optimal levels. Run 25 also produced similar high yields of reducing sugars (3.07 mg/mL), with the only difference being that the feed percentage was at its optimal level (1%) and the incubation temperature was at its low level (55 °C). Analysis of Variance (ANOVA) was performed to determine the *p*-values. The model was significant (*p* ≤ 0.05) for all enzymatically treated feed samples. Table 2 shows the results for the starter feed treatment with crude *T. harzianum* xylanase, the interactions between the feed loading and incubation time as well as the incubation time and incubation temperature (*p* ≤ 0.05), and the square terms for feed loading and incubation time (*p* ≤ 0.03), which were significant. Similarly, for the grower feed treatment, the square terms for enzyme dosage (*p* ≤ 0.0001) and incubation temperature (*p* ≤ 0.005) were significant as well as the linear terms for incubation temperature (*p* ≤ 0.004). The Pareto charts of standardization histogram graphs (Figure 1) corroborate these findings (*p* ≤ 0.05), as they crossed the p-line (cumulative% = 50%).

#### 2.1.2. Hydrolysis of Starter and Grower Feeds with Purified *T. harzianum* Xylanase

**Figure 2:**
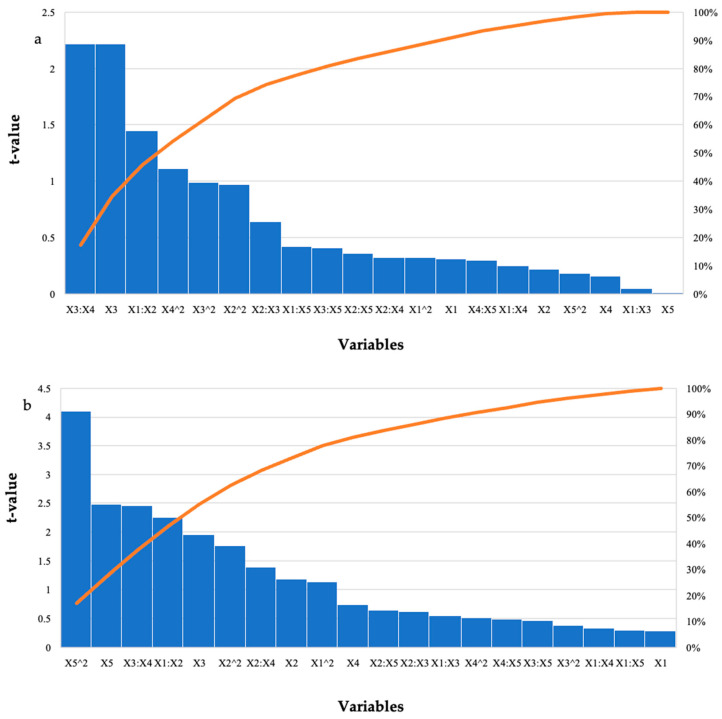
Pareto chart of standardised effects for the BBD for enzyme dosage (X1), feed loading (X2), incubation time (X3), incubation temperature (X4), and pH (X5) for the hydrolysis of (**a**) starter feed and (**b**) grower feed with purified *T. harzianum* xylanase. The orange line represents *p* = 0.05.

Hydrolysis of the starter feed by the purified *T. harzianum* xylanase resulted in the highest amount of reducing sugars in run 12 (2.81 mg/mL) at the optimal enzyme dosage and pH with the other variables at their high levels (Table 1). There were other runs that produced similar high yields; however, these were significantly different (*p* ≤ 0.05). For the grower feed, run 24 (2.41 mg/mL) with all variables at their optimal levels resulted in the highest amount of reducing sugars. Run 12 also resulted in similar yields (2.31 mg/mL) with feed loading, incubation time, and temperature at their high levels. Table 3 represents the ANOVA results for the hydrolysis of the starter and grower feeds by the purified *T. harzianum* xylanase, the interactions between the enzyme dosage and incubation time, as well as the incubation time and incubation temperature. The linear terms for time were significant (*p*-values were 0.05, 0.04, and 0.04), respectively. For treatment of the grower feed; the square (*p* ≤ 0.0003) and linear terms (*p* ≤ 0.02) for pH; as well as the interactions between the enzyme dosage and feed (*p* ≤ 0.03); and time and temperature (*p* ≤ 0.02) were significant. The Pareto charts of standardization histogram graphs (Figure 2) also showed that those terms were significant (*p* ≤ 0.05), as they crossed the p-line (cumulative% = 50%).

#### 2.1.3. Hydrolysis of Starter and Grower Feeds with Recombinant XT6 Xylanase

**Figure 3:**
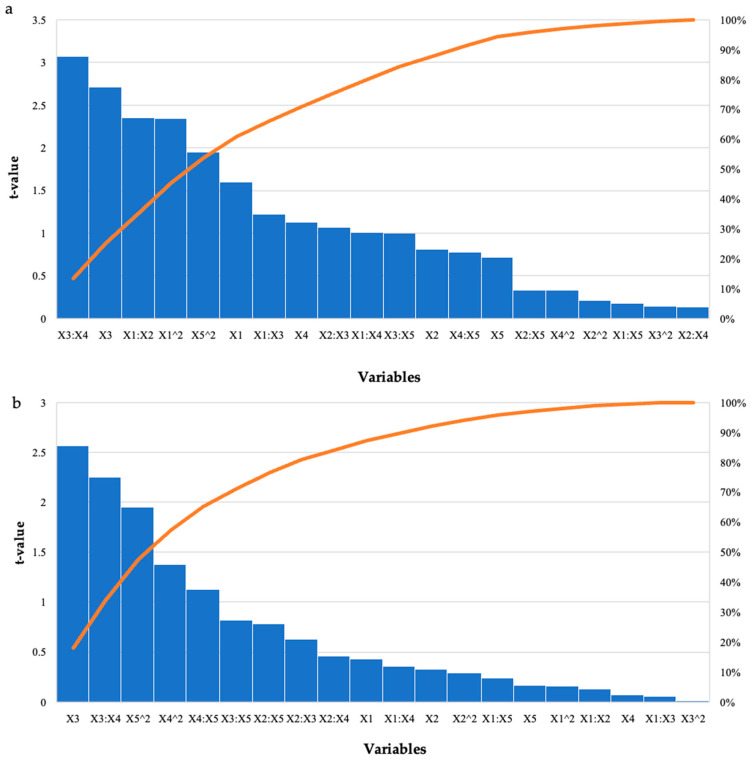
Pareto chart of standardised effects for the BBD for enzyme dosage (X1), feed loading (X2), incubation time (X3), incubation temperature (X4), and pH (X5) for the hydrolysis of (**a**) starter feed and (**b**) grower feed with purified recombinant XT6 xylanase. The orange line represents *p* = 0.05.

The run that had the highest effect on starter feed was run 12 (2.98 mg/mL), with enzyme dosage and pH at their optimal levels and the other variables at their high levels (Table 1). For the grower feed, run 12 (2.79 mg/mL) had enzyme dosage and pH at their optimal levels, and the other variables at their high levels. There were other runs that produced similar high yields; however, these were significantly different (*p* ≤ 0.05). Overall, higher levels of reducing sugars were obtained for the starter feed hydrolysis by all three enzymes compared to the grower feed. Hydrolysis of the starter feed with the crude *T. harzianum* xylanase produced the highest yield of reducing sugars (8.05 mg/mL). The lowest yield (2.41 mg/mL) was obtained for the grower feed hydrolysed by purified *T. harzianum* xylanase. Table 4 shows that the interactions between the enzyme dosage and feed loading, as well as incubation time and temperature, and the linear terms for time were significant as *p*-values were 0.0004, 0.03, and 0.01, respectively. The square terms for the enzyme dosage (*p* ≤ 0.03) and incubation temperature (*p* ≤ 0.05) were also significant. For the grower feed, the interaction between incubation time and temperature (0.03) and the linear terms (*p* ≤ 0.02) for the incubation time were significant, as well as the square term for pH (*p* ≤ 0.05). The Pareto charts of standardization histogram graphs (Figure 3) also showed that those terms were significant (*p* ≤ 0.05), as they crossed the p-line (cumulative% = 50%).

#### 2.1.4. Interaction of Variables for Feed Hydrolysis

The relationship between the responses and the parameters for feed hydrolysis generated by the quadratic model and the optimum level of each variable were studied using three-dimensional (3D) response surface plots (Figure 4, Figure 5, Figure 6, Figure 7 and Figure 8) (Supplementary Figures S1–S6), where the *z*-axis refers to reducing sugars versus any two variables, whilst the other variables are at their optimal levels 3.

##### Effect of the Crude *T. harzianum* Xylanase on Reducing Sugar Yield Following Hydrolysis of Starter and Grower Chicken Feeds

**Figure 4:**
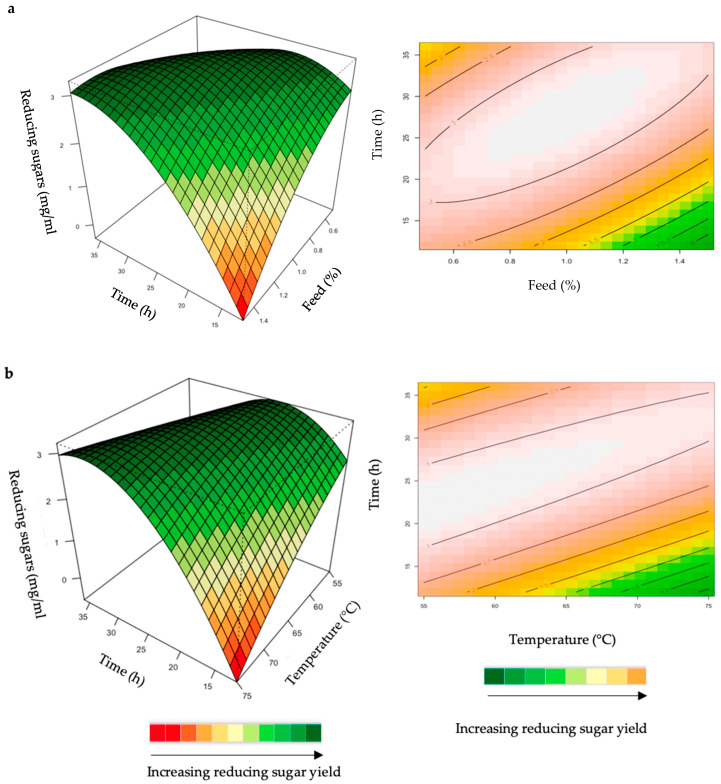
3D-response surface plots and contour plots of the combined effects of feed loading and incubation time (**a**) and incubation time and temperature (**b**) on the yield of reducing sugars from the hydrolysis of starter chicken feed by the crude *T. harzianum* xylanase.

The hydrolysis of the xylan in chicken feed can be influenced by the feed loading, enzyme dosage, incubation time, incubation temperature, and pH. The interactive effects of the variables were analysed for hydrolysis of the starter and grower chicken feeds by the crude *T. harzianum* xylanase (Figure 4a,b). For this analysis, the other parameters were kept constant at their zero (optimal) levels. The mutual interaction of the variables (feed loading: incubation time; incubation time: incubation temperature) were significant (*p* ≤ 0.05), indicating that there is a synergistic interaction favouring the production of reducing sugars by the crude *T. harzianum* xylanase from starter chicken feed. The highly elliptical response surface plot in Figure 4a shows the that highest reducing sugars yield (3 mg/mL) was produced when both variables, feed loading and incubation time, were high. Chapa et al. [29] also obtained similar results, demonstrating that as the incubation time increased there was an increase in reducing sugars. Ai et al. [30] reported 3.9 mg/mL of reducing sugars from pretreated corncobs hydrolysed for 24 h by the *Streptomyces olivaceoviridis* xylanase. Feed loading also plays an important role in enzymatic hydrolysis [29]. Increasing the concentration of feed showed a substantial increase in reducing sugars, while decreasing the feed loading from 1.0% to 1.4% decreased the yield of reducing sugars. It is clear from Figure 4a that a higher feed loading (>1.0%) does not enhance the yield of reducing sugars. The lower yield of reducing sugars from higher feed loads could be attributed to the reduced availability of water in the aqueous medium. This trend was also observed by Yoon et al. [31]. Figure 4b shows a high yield at low incubation temperatures and times. Temperature is one of the most important parameters for enzyme activity. The optimization of reaction temperature was necessary to achieve optimal functioning of the enzyme in the provided conditions because of the well-established facts of enzyme inhibition at lower temperatures and enzyme inactivation at higher temperatures [32]. Figure 4b shows that the yield of reducing sugars was significantly higher at 55 °C and an incubation time of approximately 25 h. The production of reducing sugars was reduced at 70–75 °C, which may be due to inactivation of the enzyme at these higher temperatures and longer incubation times. The interactions of the variables for the hydrolysis of grower feed by the crude *T. harzianum* xylanase were not significant (*p* ≤ 0.05) (Supplementary Figures S1 and S2).

##### Effect of the Purified *T. harzianum* Xylanase on Reducing Sugar Yield Following Hydrolysis of Starter and Grower Chicken Feeds

**Figure 5:**
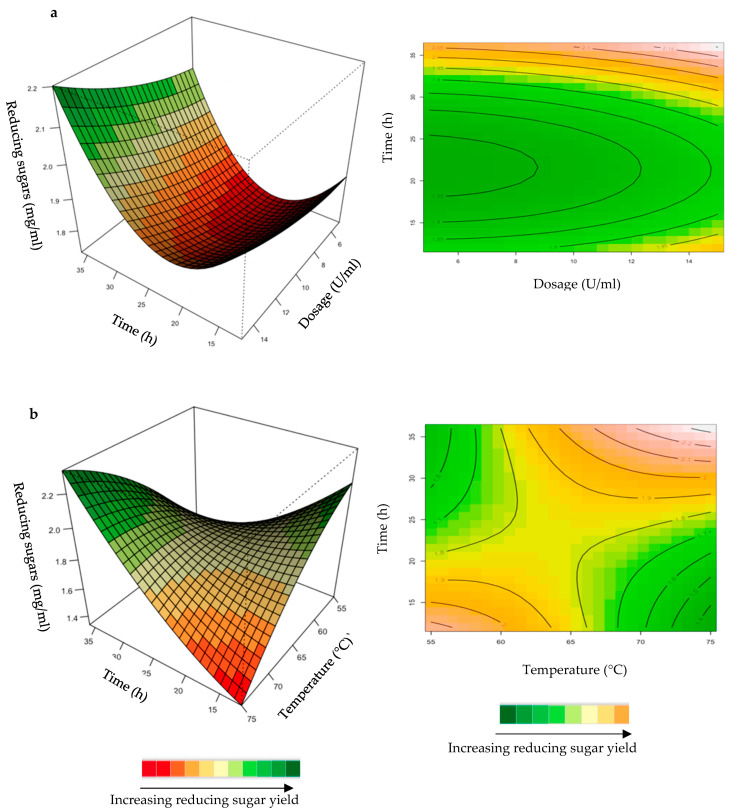
3D-response surface plots and contour plots of the combined effects of enzyme dose and incubation time (**a**) and incubation time and temperature (**b**) on the yield of reducing sugars from starter chicken feed hydrolysed by the purified *T. harzianum* xylanase.

The mutual interaction of the variables (enzyme dosage: incubation time; incubation time: temperature) was significant (*p* ≤ 0.05), indicating that there is synergistic interaction favouring the production of reducing sugars by the activity of the purified *T. harzianum* xylanase on starter chicken feeds. Enzyme dose plays a significant role in increasing the reducing sugars and XOS yield [33]. Enzyme doses in the range of 5–15 U/mL were used in the present study. Enhanced yield of reducing sugars was obtained for long incubation times and high enzyme doses (Figure 5a). The contour plot showed that an incubation time of approximately 30 h and a 13 U/mL enzyme dosage resulted in the highest yield of reducing sugars. Yang et al. [34] observed that an increase in xylanase dose from 5 to 10 U/mL increased the reducing sugar to 12 g/L from 11 g/L after 24 h of incubation in their experiments. Enzymes can be more effective after a pre-treatment of the substrate, since this increases the accessibility of the active sites of the substrate to the enzyme. The decreased effectiveness of enzyme activity on untreated substrates could be attributed to the location of the hydrolysable xylans, which are usually located at the periphery of the particles of substrates [33]. The interaction between incubation time and temperature (Figure 5b) resulted in the highest yield of reducing sugars at high (75 °C) temperatures and prolonged incubation times (35 h). 

**Figure 6:**
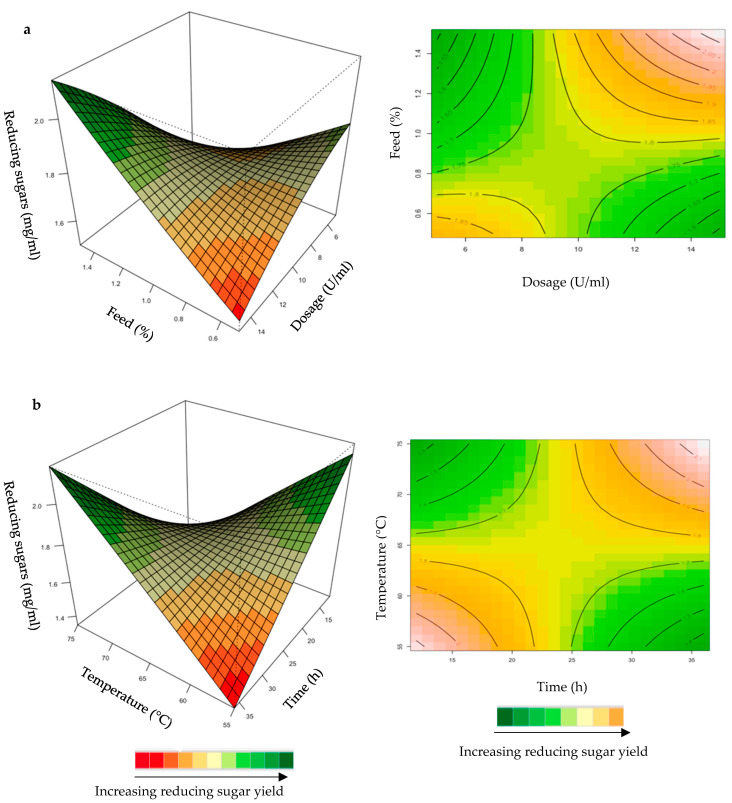
3D-response surface plots and contour plots of the combined effects of enzyme dose and feed loading (**a**) and incubation time and temperature (**b**) on the yield of reducing sugars from grower chicken feed hydrolysed by the purified *T. harzianum* xylanase.

For the optimization of reducing sugars from hydrolysis of grower chicken feed, the yield was enhanced at high dosage and feed loading (Figure 6a). For the interaction between time and temperature (Figure 6b), the reducing sugars yield was enhanced at long incubation times and high temperatures. The positive effects of high temperatures on the production of reducing sugars is the dissolution of xylan, the prevention of microbial contamination, and an increase in the reaction rate [35]. The interactions of the other variables were insignificant (Supplementary Figures S3 and S4).

##### Effect of the Recombinant XT6 Xylanase on Reducing Sugar Yield Following Hydrolysis of Starter and Grower Chicken Feeds

**Figure 7:**
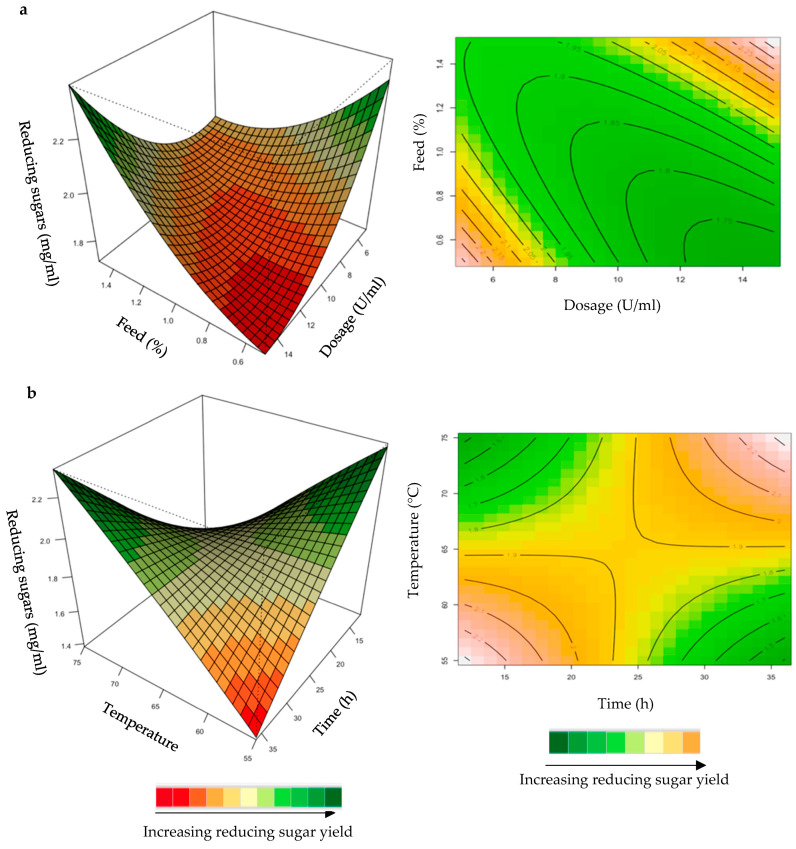
3D-response surface plots and contour plots of the combined effects of dose and feed loading (**a**) and incubation time and temperature (**b**) on the yield of reducing sugars from the starter chicken feed hydrolysed by the purified recombinant XT6.

The mutual interaction of the variables (enzyme dose: feed loading; incubation time: temperature) were significant (*p* ≤ 0.05), indicating that there is synergistic interaction favouring the production of reducing sugars by the purified recombinant XT6 on starter feed. An enhanced yield of reducing sugars was evident at high enzyme doses and feed loading (Figure 7a). At high incubation temperatures and long incubation times, the yield of reducing sugars was enhanced (Figure 7b). Li et al. [36] reported a *Streptomyces* spp. T7 which was used to produce XOSs from corncob xylan at 60 °C, with the highest yield of reducing sugars. Khangwal et al. [21] also reported a recombinant xylanase, SipoEnXyn10 (*Streptomyces ipomoeae* cloned and expressed in *E. coli*), which was used to produce XOS from beechwood xylan at 65 °C with the highest yield of reducing sugars.

**Figure 8:**
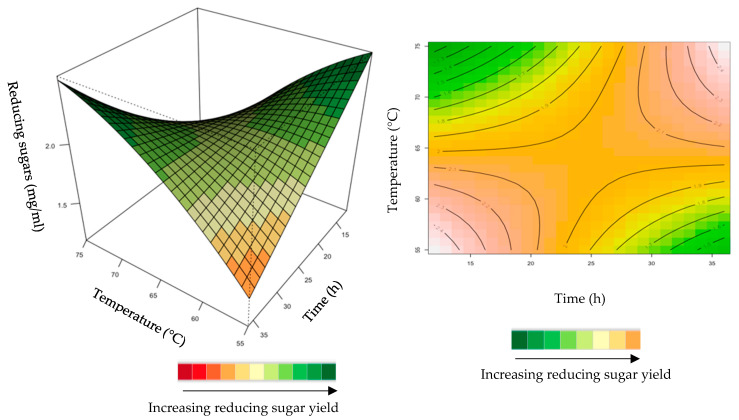
3D-response surface plot and contour plot of the combined effects of incubation time and temperature on the yield of reducing sugars from the grower chicken feed hydrolysed by the purified recombinant XT6.

The interactive effect of time and temperature were examined, and the results are illustrated in Figure 8. The mutual interaction of these variables (time: temperature) was significant (*p* > 0.05), indicating that there is synergistic interaction favouring the production of reducing sugars by the purified recombinant XT6 on grower feed. Both high (35 h and 75 °C) and low (15 h and 55 °C) levels in BBD enhanced the yield of reducing sugars.

### 2.2. Thin Layer Chromatography (TLC) and High Performance Liquid Chromatography (HPLC) Analysis of Feed Hydrolysis Products

**Figure 9:**
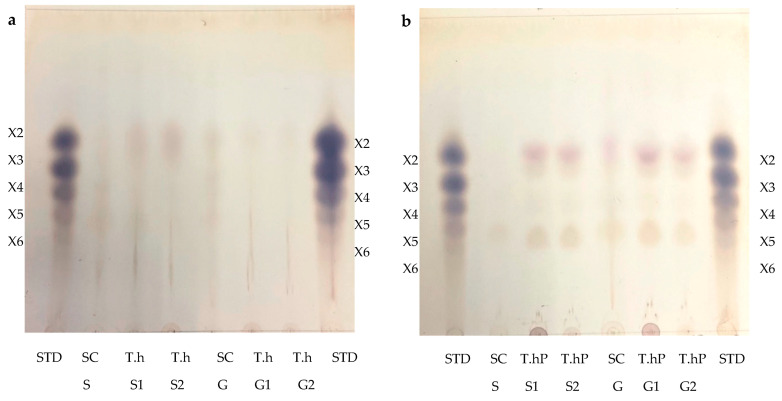

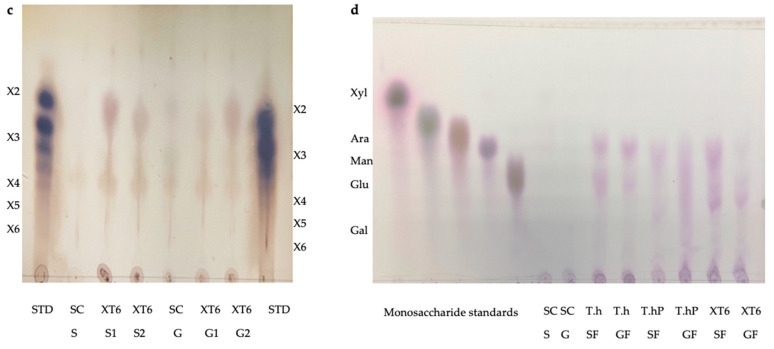
Thin Layer Chromatography (TLC) profile of XOS produced from chicken feed hydrolysis by the crude (**a**) and purified (**b**) *T. harzianum* xylanases and the recombinant XT6 xylanase (**c**). (**d**) shows the TLC profile of the monosaccharides resulting from chicken feed hydrolysis by the three enzyme preparations. STD—Xylooligosaccharides standards, SC (S)—substrate control (no xylanase) starter feed, SC (G)–Substrate control (no xylanase) grower feed, T.h S1—starter feed with crude *T. harzianum* xylanase (sample 1), T.h S2—starter feed with crude *T. harzianum* xylanase (sample 2), T.h G—grower feed with crude *T. harzianum* xylanase (sample 1) and T.h G2—grower feed with crude *T. harzianum* xylanase (sample 2). T.hP S1—starter feed with purified *T. harzianum* xylanase (sample 1), T.hP S2—starter feed with purified *T. harzianum* xylanase (sample 2), T.hP G1–grower feed with purified *T. harzianum* xylanase (sample 1) and T.hP G2—grower feed with purified *T. harzianum* xylanase (sample 2). X2—Xylobiose; X3—Xylotriose; X4—Xylotetraose; X5—Xylopentaose; X6—Xylohexaose. Monosaccharide standards; Xyl—xylose, Ara–arabinose, Man—mannose, Glu—glucose, and Gal—galactose. T.h SF—starter feed with crude *T. harzianum* xylanase, T.h GF—grower feed with crude *T. harzianum* xylanase, T.hP SF—starter feed with purified *T. harzianum* xylanase, T.hP GF—grower feed with purified *T. harzianum* xylanase, XT6 SF—recombinant XT6 xylanase with starter feed and XT6 GF–recombinant XT6 xylanase with grower feed.

The TLC was performed to visualize the monosaccharides/XOS and the degree of polymerization (DP) of the XOS produced following the hydrolysis of the local chicken feeds by the three enzyme preparations (Figure 9a–d). After the optimal hydrolysis treatments, 2.9 U/mL, 8.65 U/mL, and 3.63 U/mL reducing sugars were produced from starter feed hydrolysed by the crude *T. harzianum* xylanase, purified *T. harzianum* xylanase, and recombinant XT6 xylanase, respectively. Hydrolysis of the grower chicken feed produced 2.5 U/mL, 3.96 U/mL, and 3.60 U/mL reducing sugars by the crude fungal xylanase, purified fungal xylanase, and recombinant xylanase, respectively. The TLC analysis indicated the production of XOS of DP 2–6 (equivalent to X2–X6) in the enzymatic reactions. The substrate controls displayed some faint spots that corresponded to those of the hydrolysed samples. This may have been due to their breakdown during the termination of the reaction (heating at 100 °C). Figure 9d shows the monosaccharides glucose and galactose following chicken feed hydrolysis. One of the attractive features of the process was the production of only XOS and no xylose. The absence of xylose and the predominant production of xylobiose suggest a unique specificity and catalytic mechanism of the thermophilic xylanase. This indicates that the enzyme has a higher affinity for cleaving the glycosidic bonds at specific positions within the xylan substrate, resulting in the release of xylobiose as the primary product. The absence of xylose in our study was beneficial because previous research has indicated that xylose production can hinder the production of XOS [37]. Hegazy et al. [38] also reported non-competitive end product inhibition by xylose of a *G. stearothermophilus* derived xylanase, XT6.

**Figure 10:**
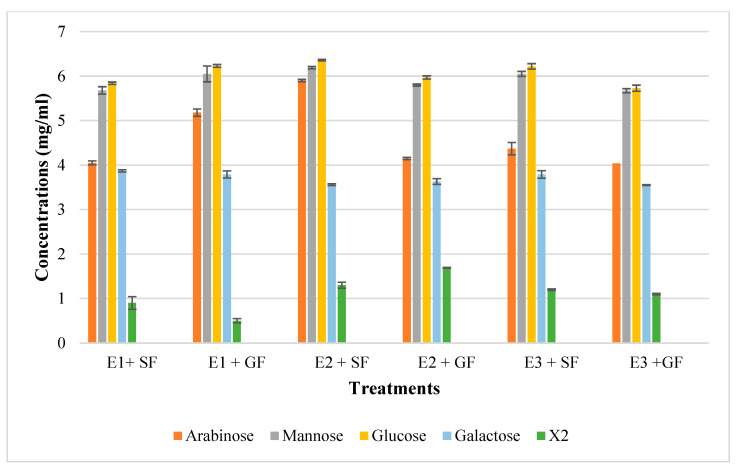
The High-Performance Liquid Chromatography (HPLC) profile of monosaccharides and xylooligosaccharides resulting from chicken feed hydrolysis. E1 + SF Crude *T. harzianum* xylanase + starter chicken feed, E1 + G—crude *T. harzianum* xylanase + grower chicken feed, E2 + S—purified *T. harzianum* xylanase + starter chicken feed, E2 + G—purified *T. harzianum* xylanase + grower chicken feed, E3 + S—purified recombinant XT6 xylanase + starter chicken feed and E3 + G—purified recombinant XT6 xylanase + grower chicken feed. Data points represent the mean values ± SD (n = 3).

In addition to glucose and galactose observed on TLC (Figure 9d), hydrolysis of chicken feeds by the three enzyme preparations also produced mannose evident in HPLC chromatograms (Figure 10). Xylobiose (X2) was the only XOS observed by HPLC (Figure 10). Khangwal et al. [21] observed xylobiose as the major product from corncobs and *Moso bamboo.* The hydrolysis of grower chicken feed by purified *T. harzianum* xylanase produced the highest concentration of xylobiose and the lowest concentration was observed by the hydrolysis of grower feed hydrolysed by the crude *T. harzianum* xylanase. Overall, hydrolysis using the purified *T. harzianum* xylanase resulted in higher monosaccharides and xylobiose concentrations than the crude *T. harzianum* xylanase and recombinant XT6 xylanase. The yield of XOS with DP 3 and higher could not be measured due to the unresolved HPLC peaks. However, the spots for X3, X4, and X5 were noted to be predominant as shown on TLC chromatograms.

Lately, XOS (particularly xylobiose) has attracted interest as an effective prebiotic that has beneficial effects on animal and human digestion [39]. Xylanases are desirable for XOS production from biomass hydrolysis. TLC analysis revealed that the hydrolysis of xylan biomass produced short-chain (DP 2–6) XOS (Figure 9). Similar results were obtained in previous reports on xylanases [40,41]. The production of XOS of similar DP at moderate temperatures highlights the suitability of xylanases for the bioprocessing industries that are preferably performed with less (heat) energy input. Nonetheless, the HPLC-based estimation of XOS yield by xylanases does not include the DP 3 and higher oligosaccharides that are evident in TLC chromatograms (Figure 9a–c). XOS is reported to have the capability of aiding in the proliferation of the population of beneficial gut microflora [42,43]. Further, the XOS of this DP range (2–6) has enormous intestinal-health potential and anti-cancerous prospects [7]. Hydrolysis of starter and grower feeds produced XOS that transitioned between the standards on TLC (Figure 9). This observation may have been due to the substitution of the arabinoxylan in the feeds, which led to the formation of a suspension of feeds in the buffer and, thus, did not result in the release of soluble xylans [44]. 

## 3. Materials and Methods

### 3.1. Feed Samples and Enzymes

Chicken starter and grower feed substrates were obtained from Rhodes University, Grahamstown, Eastern Cape. According to Biasato et al. [45], the feeds for monogastric animals such as chickens and pigs in South Africa primarily consist of corn as the main energy source and soybean as the main protein source. Studies by El-Deek et al. [46] and Saleh and Watkins [47] have reported that the formulations of starter and grower feeds for broilers often vary in terms of the ratio of corn to soybean. It was observed that starter feeds generally contain more soybean and less corn compared to grower feeds [44]. Table 5 represents the composition of the feeds used in this study. A crude and purified *T. harzianum* xylanase was previously studied and included in this study [19,25]. A recombinant XT6 xylanase previously optimized and purified [26] and was included in this study. 

### 3.2. Optimization of the Hydrolysis of Feed Using the Crude and Pure Fungal T. harzianum and Pure Recombinant XT6 Xylanases

The crude and purified *T. harzianum* xylanases and purified recombinant XT6 xylanase were used in this study to hydrolyse xylan in starter and grower chicken feeds. The RSM using the BBD was used to study the influence of five variables on the hydrolysis of chicken feeds by xylanases and to statistically determine the optimum combination of enzyme dosage, feed loading, incubation time, pH, and incubation temperature for enhanced hydrolysis, which was achieved by monitoring reducing sugars (mg/mL) as the endpoint. This was achieved using the 3,5-Dinitrosalicylic acid (DNS) assay. The main interactions and the quadratic effects of the variables on enzymatic hydrolysis of the feed were also assessed, and a five-factor, three-level design was applied to investigate the quadratic response surfaces and construct secondary polynomial models. Each variable was coded and run at three independent levels, (−), (0), and (+) levels. The significant relationships in the model were assessed and all the statistical analyses were carried out using R Studio software (http://www.R-project.org/ (accessed on 15 March 2023)) [48]. The effect of each factor and their interactions on the dependent variables was assessed by the two-way Analysis of variance (ANOVA) technique [19,49]. The optimization data were analysed to determine the regression coefficients to arrive at the regression equation. The regression model containing coefficients, including the linear and quadratic effect of factors and the linear effect of interactions, was assumed to describe relationships between response (Y) and the experimental factors (X1, X2, X3, X4, and X5). 

The second-order polynomial equation is shown below in Equation (1):

(1) Y=β0+∑βi Χ1+∑βii Χ2+∑βij Χ1×2

where β0 is the constant coefficient, βi is the linear coefficient of main factors, βii is the quadratic coefficient for main factors, and βij is the second-order interaction coefficient. The response variable was assigned at low and high of the observed values for the desirability of 0 and 1, respectively, to obtain the overall desirability [29].

### 3.3. Chromatographic Analysis of Hydrolysed Products

The qualitative and quantitative analyses of monosaccharides and XOS resulting from the feed hydrolysis were carried out using 2 μL aliquots from each hydrolysate for TLC and HPLC. The hydrolysate samples were applied to Silica Gel 60 F254 TLC plates (Merck, Darmstadt, Germany), which were then developed in a 1-butanol: acetic acid: water (2:1:1, *v*/*v*/*v*) mobile phase. The plates were left to air dry for 1 h and were then stained by soaking in Molisch’s Reagent (0.3% (*w*/*v*) α-naphthol dissolved in a sulfuric acid: methanol solution (5:95, *v*/*v*)). The sugars developed on the plates were finally visualized by heating the plates at 110 °C in an oven (Heraeus B6120 Incubator, Gemini BV, Apeldoorn, The Netherlands) for 15 min. An XOS standard containing a mixture of xylobiose (X2), xylotriose (X3), xylotetraose (X4), xylopentaose (X5), and xylohexaose (X6)) was obtained from Professor Kugen Perumal at Durban University of Technology. Monosaccharide standards (xylose, arabinose, mannose, glucose, and galactose) were purchased from Sigma, Aldrich, Modderfontein, South Africa). The yield of monosaccharides and XOS were estimated by HPLC. The supernatant fractions from the hydrolysates were filtered using a 0.2 μm filter. The XOS and monosaccharides in the samples were quantified with a Shimadzu RID-20A HPLC system (Shimadzu Scientific Instruments, Southern California, CA, USA) using a BioRad Aminex HPX-87H column (Bio-Rad, Transgenomic, Inc., Omaha, NE, USA) at 50 °C with a mobile phase of 5 mM H_2_SO_4_ and a flow rate of 0.5 mL/min and samples were analysed with a refractive index (RI) detector. 

## 4. Conclusions

The present study established the potential of native *T. harzianum* and recombinant *G. strearothermophilus* xylanases for the enhancement of the hydrolysate product and the production of XOS from starter and grower chicken feeds. Starter feed hydrolysis resulted in higher yields of reducing sugars compared to grower feed. Overall, the purified *T. harzianum* xylanase resulted in a higher yield of reducing sugars compared to the crude *T. harzianum* xylanase and recombinant XT6 xylanase. The RSM efficiently optimized the yield of reducing sugars and quantified the interactive effects of the significant variables. The xylanases were efficient in releasing short-chain XOSs (xylobiose, xylotriose, xylotetraose, and xylopentaose) and monosaccharides (glucose, galactose, and mannose), with xylobiose being the dominant product. This shows interesting prospects for future studies using XOS as prebiotics in the feed industry and to reduce viscosity and improve the gut microbiota.

## Figures and Tables

**Table 1:** Experimental design for the Box Behnken Design (BBD) model for five independent variables tested for reducing sugar production from two chicken feed types.

	Variable Level					Reducing Sugars (mg/mL)					
Run Order	X1 (U/mL)	X2 (%)	X3 (h)	X4 (°C)	X5 (pH)	Starter Chicken Feed			Grower Chicken Feed		
						Crude *T. harzianum* Xylanase	Purified *T. harzianum* Xylanase	Recombinant XT6 Xylanase	Crude *T. harzianum* Xylanase	Purified *T. harzianum* Xylanase	Recombinant XT6 Xylanase
1	**5 (−)**	**0.5 (−)**	**24 (0)**	**65 (0)**	5 (0)	3.16	0.82	2.36	3.21	2.09	2.37
2	15 (+)	0.5 (−)	24 (0)	65 (0)	5 (0)	3.10	1.90	1.68	2.92	1.59	2.17
3	5 (−)	1.5 (+)	24 (0)	65 (0)	5 (0)	2.78	1.99	2.12	2.56	1.73	2.62
4	15 (+)	1.5 (+)	24 (0)	65 (0)	5 (0)	1.95	2.22	2.46	2.96	2.21	2.34
5	5 (−)	1 (0)	12 (−)	65 (0)	5 (0)	2.02	2.23	2.10	2.85	1.68	2.20
6	15 (+)	1 (0)	12 (−)	65 (0)	5 (0)	2.74	2.25	2.57	2.84	1.93	2.14
7	5 (−)	1 (0)	36 (+)	65 (0)	5 (0)	2.86	2.15	2.03	2.69	1.49	2.22
8	15 (+)	1 (0)	36(+)	65 (0)	5 (0)	3.23	2.21	1.97	2.50	1.97	2.12
9	10 (0)	0.5 (−)	12 (−)	55 (−)	5 (0)	2.60	1.87	2.18	2.46	2.00	2.16
10	10 (0)	1.5 (+)	12 (−)	55 (−)	5 (0)	2.82	2.13	2.05	2.51	2.10	2.45
11	10 (0)	0.5 (−)	36 (+)	75 (+)	5 (0)	2.21	2.11	2.27	2.40	2.17	2.53
12	10 (0)	1.5 (+)	36 (+)	75 (+)	5 (0)	2.93	**2.81**	**2.98**	2.83	2.31	**2.79**
13	10 (0)	1 (0)	24 (0)	55 (−)	4 (−)	2.74	2.05	2.17	2.79	1.85	2.62
14	10 (0)	1 (0)	24 (0)	75 (+)	4 (−)	2.68	2.00	1.97	2.59	1.73	1.99
15	10 (0)	1 (0)	24 (0)	55 (−)	6 (+)	2.38	1.68	1.67	2.57	1.55	1.81
16	10 (0)	1 (0)	24 (0)	75 (+)	6 (+)	2.56	1.80	1.81	2.60	1.65	1.92
17	10 (0)	0.5 (−)	24 (0)	65 (0)	4 (−)	2.60	1.88	1.71	2.28	1.29	1.94
18	10 (0)	1.5 (+)	24 (0)	65 (0)	4 (−)	2.52	1.78	1.71	2.46	1.03	1.69
19	10 (0)	0.5 (−)	24 (0)	65 (0)	6 (+)	2.50	1.80	1.57	2.28	1.65	1.73
20	10 (0)	1.5 (+)	24 (0)	65 (0)	6 (+)	2.08	1.91	1.72	2.31	1.68	1.99
21	10 (0)	1 (0)	24 (0)	65 (0)	5 (0)	1.92	2.01	1.86	1.95	1.81	1.41
22	10 (0)	1 (0)	24 (0)	65 (0)	5 (0)	2.32	1.88	1.92	1.82	1.92	2.44
23	10 (0)	1 (0)	24 (0)	65 (0)	5 (0)	2.21	2.13	1.94	1.77	1.91	1.95
24	10 (0)	1 (0)	24 (0)	65 (0)	5 (0)	2.18	2.27	2.10	2.46	**2.41**	2.11
25	5 (−)	1 (0)	24 (0)	55 (−)	5 (0)	3.03	1.78	2.20	3.07	1.77	1.92
26	15 (+)	1 (0)	24 (0)	55 (−)	5 (0)	2.66	1.59	1.64	2.63	1.60	1.73
27	5 (−)	1 (0)	24 (0)	75 (+)	5 (0)	2.56	1.63	1.85	2.45	1.89	1.75
28	15 (+)	1 (0)	24 (0)	75 (+)	5 (0)	2.51	1.60	1.73	2.22	1.87	1.80
29	5 (−)	1 (0)	24 (0)	65 (0)	4 (−)	2.33	1.86	1.87	2.16	1.25	1.65
30	15 (+)	1 (0)	24 (0)	65 (0)	4 (−)	2.47	1.91	1.86	2.36	1.63	1.51
31	5 (−)	1 (0)	24 (0)	65 (0)	6 (+)	2.41	1.90	1.89	2.22	1.51	1.89
32	15 (+)	1 (0)	24 (0)	65 (0)	6 (+)	2.05	1.71	1.80	2.13	1.76	1.90
33	10 (0)	0.5 (−)	12 (−)	65 (0)	5 (0)	2.36	1.78	1.74	2.03	1.70	2.13
34	10 (0)	1.5 (+)	12 (-)	65 (0)	5 (0)	−5.86	1.71	1.89	1.76	1.85	2.24
35	10 (0)	0.5 (−)	36 (+)	65 (0)	5 (0)	2.28	1.90	1.69	1.58	1.72	1.91
36	10 (0)	1.5 (+)	36(+)	65 (0)	5 (0)	2.48	2.16	1.92	2.10	1.89	2.48
37	10 (0)	0.5 (−)	24 (0)	55 (−)	5 (0)	2.97	1.80	1.84	2.89	1.96	2.03
38	10 (0)	1.5 (+)	24 (0)	55 (−)	5 (0)	4.45	1.60	2.07	2.56	1.79	1.92
39	10 (0)	0.5 (−)	24 (0)	75 (+)	5 (0)	2.51	1.52	1.79	2.31	1.35	1.59
40	10 (0)	1.5 (+)	24 (0)	75 (+)	5 (0)	2.61	1.49	1.66	2.22	1.91	1.36
41	10 (0)	1 (0)	12 (−)	65 (0)	4 (−)	2.49	1.60	1.67	2.24	1.71	1.93
42	10 (0)	1 (0)	36 (+)	65 (0)	4 (−)	2.40	1.80	1.69	2.48	1.77	1.60
43	10 (0)	1 (0)	12 (−)	65 (0)	6 (+)	2.07	1.77	1.67	2.21	1.74	1.61
44	10 (0)	1 (0)	36 (+)	65 (0)	6 (+)	2.41	1.73	1.64	2.22	1.60	1.83
45	10 (0)	1 (0)	24 (0)	65 (0)	5 (0)	**8.05**	1.60	1.65	1.98	1.75	2.03
46	10 (0)	1 (0)	24 (0)	65 (0)	5 (0)	4.91	1.51	1.80	1.81	1.75	2.16
47	10 (0)	1 (0)	24 (0)	65 (0)	5 (0)	1.90	1.52	1.74	1.62	1.89	2.29
48	10 (0)	1 (0)	24 (0)	65 (0)	5 (0)	7.84	1.95	2.11	1.96	2.18	2.33

**Table 2:** Analysis of Variance (ANOVA) and regression coefficients of the response surface quadratic model for the response variables for optimizing chicken feed hydrolysis by crude *T. harzianum* xylanase.

Variable	Estimate		Std. Error		*t* Value		*p*-Value	
	Starter Feed	Grower Feed	Starter Feed	Grower Feed	Starter Feed	Grower Feed	Starter Feed	Grower Feed
Model	−23.24	27.40	49.43	8.95	−0.47	3.06	0.05 *	0.005 *
Enzyme Dosage (U/mL)	0.48	−0.45	1.68	0.30	0.28	−1.47	0.78	0.15
Feed Loading (%)	14.41	−2.59	14.11	2.55	1.02	−1.02	0.32	0.32
Incubation Time (h)	−0.61	−0.01	0.77	0.14	−0.79	−0.01	0.43	0.99
Incubation Temperature (°C)	0.30	−0.53	0.94	0.17	0.32	−3.10	0.75	0.004 *
pH	6.51	−1.70	9.13	1.65	0.71	−1.03	0.48	0.31
Enzyme Dosage (U/mL): Feed Loading (%)	−0.08	0.07	0.37	0.07	−0.21	1.03	0.84	0.31
Enzyme Dosage (U/mL): Time (h)	−0.01	−0.01	0.02	0.02	−0.10	−0.26	0.92	0.80
Enzyme Dosage (U/mL): Temperature (°C)	0.01	−0.01	0.02	0.01	0.09	0.32	0.93	0.75
Enzyme Dosage (U/mL): pH	−0.02	−0.01	0.18	0.03	−0.14	−0.43	0.89	0.67
Feed Loading (%): Incubation Time (h)	0.26	0.02	0.13	0.02	2.07	1.06	0.03 *	0.30
Feed Loading (%): Incubation Temperature (°C)	−0.18	0.00	0.15	0.02	−1.18	0.03	0.25	0.98
Feed Loading (%): pH	−0.17	−0.07	1.84	0.33	−0.09	−0.22	0.93	0.83
Incubation Time (h): Incubation Temperature (°C)	0.01	−0.01	0.01	0.01	1.18	−0.15	0.05 *	0.88
Incubation Time (h): pH	0.01	−0.01	0.08	0.01	0.12	−0.34	0.91	0.73
Temperature (°C): pH	0.01	0.01	0.09	0.02	0.06	0.33	0.95	0.74
Enzyme Dosage (U/mL)^2	−0.02	0.02	0.02	0.01	−0.69	4.41	0.50	0.0001 *
Feed Loading (%)^2	−4.12	0.81	2.43	0.44	−1.70	1.86	0.04 *	0.07
Incubation Time (h)^2	−0.01	0.01	0.01	0.01	−2.08	0.72	0.03 *	0.48
Incubation Temperature (°C)^2	−0.01	0.03	0.01	0.01	−0.59	3.02	0.56	0.005 *
pH^2	−0.68	0.02	0.61	0.11	−1.12	1.47	0.27	0.15

* *p* ≤ 0.05 shows significance. Lack of fit = 0.98. Lack of fit = 0.16.

**Table 3:** ANOVA and regression coefficients of the response surface quadratic model for the response variables for optimizing chicken feed hydrolysis by the purified *T. harzianum* xylanase.

Variable	Estimate		Std. Error		*t* Value		*p*-Value	
	Starter Feed	Grower Feed	Starter Feed	Grower Feed	Starter Feed	Grower Feed	Starter Feed	Grower Feed
Model	3.52	1.06	7.90	5.87	0.44	0.18	0.03 *	0.03 *
Enzyme Dosage (U/mL)	0.08	−0.06	0.27	0.20	0.31	−0.29	0.76	0.77
Feed Loading (%)	0.50	−1.99	2.26	1.68	0.22	−1.19	0.83	0.24
Incubation Time (h)	−0.27	−0.18	0.12	0.09	−2.22	−1.95	0.04 *	0.06
Incubation Temperature (°C)	0.02	−0.08	0.15	0.11	0.16	−0.74	0.87	0.47
pH	−0.015	2.69	1.46	1.08	−0.01	2.48	0.99	0.02 *
Enzyme Dosage (U/mL): Feed Loading (%)	−0.09	0.10	0.06	0.04	−1.45	2.25	0.16	0.03 *
Enzyme Dosage (U/mL): Time (h)	0.00	0.01	0.00	0.01	0.05	0.55	0.05 *	0.59
Enzyme Dosage (U/mL): Temperature (°C)	0.00	0.01	0.00	0.02	0.25	0.33	0.80	0.74
Enzyme Dosage (U/mL): pH	−0.01	−0.01	0.03	0.02	−0.42	−0.30	0.68	0.77
Feed Loading (%): Incubation Time (h)	0.01	−0.01	0.02	0.01	0.64	−0.62	0.53	0.54
Feed Loading (%): Incubation Temperature (°C)	0.01	0.02	0.02	0.01	0.32	1.39	0.75	0.18
Feed Loading (%): pH	0.11	0.14	0.2	0.22	0.36	0.65	0.72	0.52
Incubation Time (h): Incubation Temperature (°C)	0.00	0.03	0.00	0.01	2.22	2.46	0.04 *	0.02 *
Incubation Time (h): pH	−0.00	−0.04	0.01	0.02	−0.41	−0.47	0.69	0.64
Temperature (°C): pH	0.00	0.05	0.01	0.01	−0.30	0.49	0.77	0.63
Enzyme Dosage (U/mL)^2	0.00	−0.03	0.00	0.02	0.32	−1.14	0.75	0.27
Feed Loading (%)^2	−0.38	−0.50	0.39	0.29	−0.97	−1.76	0.34	0.09
Incubation Time (h)^2	0.00	−0.01	0.00	0.01	0.99	−0.38	0.33	0.71
Incubation Temperature (°C)^2	−0.00	−0.01	0.00	0.01	−1.11	−0.51	0.28	0.61
pH^2	−0.01	−0.30	0.10	0.07	−0.18	−4.10	0.86	0.0003 *

* *p* ≤ 0.05 shows significance. Lack of fit = 0.40. Lack of fit = 0.62.

**Table 4:** ANOVA and regression coefficients of the response surface quadratic model for the response variables for optimizing chicken feed hydrolysis by the purified recombinant XT6 xylanase.

Variable	Estimate		Std. Error		*t* Value		*p*-Value	
	Starter Feed	Grower Feed	Starter Feed	Grower Feed	Starter Feed	Grower Feed	Starter Feed	Grower Feed
Model	9.32	6.51	5.83	8.73	1.59	0.75	0.04 *	0.04 *
Enzyme Dosage (U/mL)	−3.17	−1.28	1.98	2.96	−1.60	−0.43	0.12	0.67
Feed Loading (%)	−1.34	−8.30	1.67	2.49	−0.81	−0.33	0.43	0.74
Incubation Time (h)	−2.48	−3.51	9.14	1.37	−2.71	−2.57	0.01 *	0.02 *
Incubation Temperature (°C)	−1.25	1.10	1.11	1.66	−1.13	0.07	0.27	0.95
pH	7.80	2.75	1.08	1.61	0.72	0.17	0.48	0.87
Enzyme Dosage (U/mL): Feed Loading (%)	1.03	−8.60	4.36	6.53	2.35	−0.13	0.03 *	0.90
Enzyme Dosage (U/mL): Time (h)	−2.21	−1.57	1.81	2.72	−1.22	−0.06	0.23	0.95
Enzyme Dosage (U/mL): Temperature (°C)	2.21	1.17	2.18	3.27	1.01	0.36	0.32	0.72
Enzyme Dosage (U/mL): pH	−3.85	7.88	2.18	3.27	−0.18	0.24	0.86	0.81
Feed Loading (%): Incubation Time (h)	1.59	1.40	1.48	2.22	1.07	0.63	0.29	0.53
Feed Loading (%): Incubation Temperature (°C)	−2.30	−1.24	1.78	2.67	−0.13	−0.46	0.90	0.65
Feed Loading (%): pH	7.25	2.54	2.18	3.27	0.33	0.78	0.74	0.44
Incubation Time (h): Incubation Temperature (°C)	4.04	4.43	1.32	1.97	3.07	2.25	0.004 *	0.03 *
Incubation Time (h): pH	−8.96	1.12	9.09	1.36	−1.00	0.82	0.92	0.42
Temperature (°C): pH	8.51	1.85	1.09	1.63	0.78	1.13	0.44	0.27
Enzyme Dosage (U/mL)^2	6.70	7.01	2.86	4.29	2.34	0.16	0.03 *	0.87
Feed Loading (%)^2	−6.10	1.24	2.86	4.29	−0.21	0.29	0.83	0.77
Incubation Time (h)^2	−7.93	−9.33	5.58	8.34	−0.14	−0.01	0.89	0.99
Incubation Temperature (°C)^2	−2.64	−1.66	8.03	1.20	−0.33	−1.38	0.05 *	0.18
pH^2	−1.40	−2.09	7.16	1.07	−1.95	−1.95	0.06	0.05 *

* *p* ≤ 0.05 shows significance. Lack of fit = 0.40. Lack of fit = 0.62.

**Table 5:** Feed composition of the starter and grower feeds for broilers.

Composition (%)	Starter Feed	Grower Feed
Methionine	0.23	0.10
Lysine	0.10	0.16
Kynofos 21 (Mono dicalcium phosphate (MDCP))	1.15	0.80
Salt	0.36	0.30
Premix	0.30	0.30
Feed lime	1.46	2.34
Maize bran	4.00	6.00
Soybean	35.00	20.00
Maize	57.40	70.00

## Data Availability

The datasets used and/or analysed during the current study are available from the corresponding author upon reasonable request. Other data generated or analysed during this study are included in this article [and its supplementary information file].

## Supplementary Materials

The supporting information can be downloaded at: https://www.mdpi.com/article/10.3390/ijms242317110/s1.
