# Supplementary material for: Optimization of Xylooligosaccharides Production by Native and Recombinant Xylanase Hydrolysis of Chicken Feed Substrates
Source: Int J Mol Sci. 2023 Dec 4;24(23):17110. doi: 10.3390/ijms242317110 (PMC10707560; doi:10.3390/ijms242317110)
Supplement: Supplementary file 1 [file ijms-24-17110-s001.zip › ijms-2708967-supplementary.pdf]

SUPPLEMENTARY FIGURES

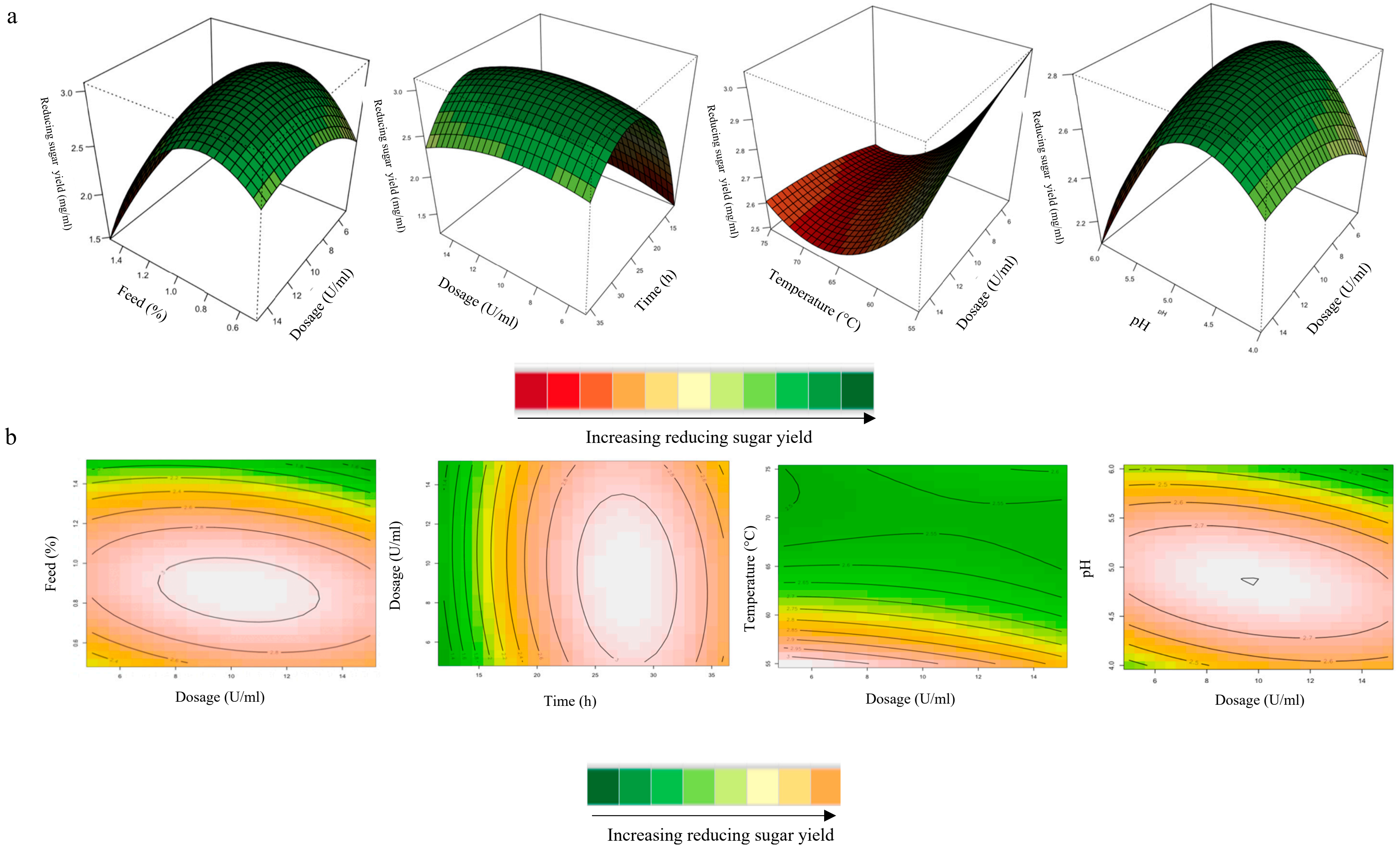

c

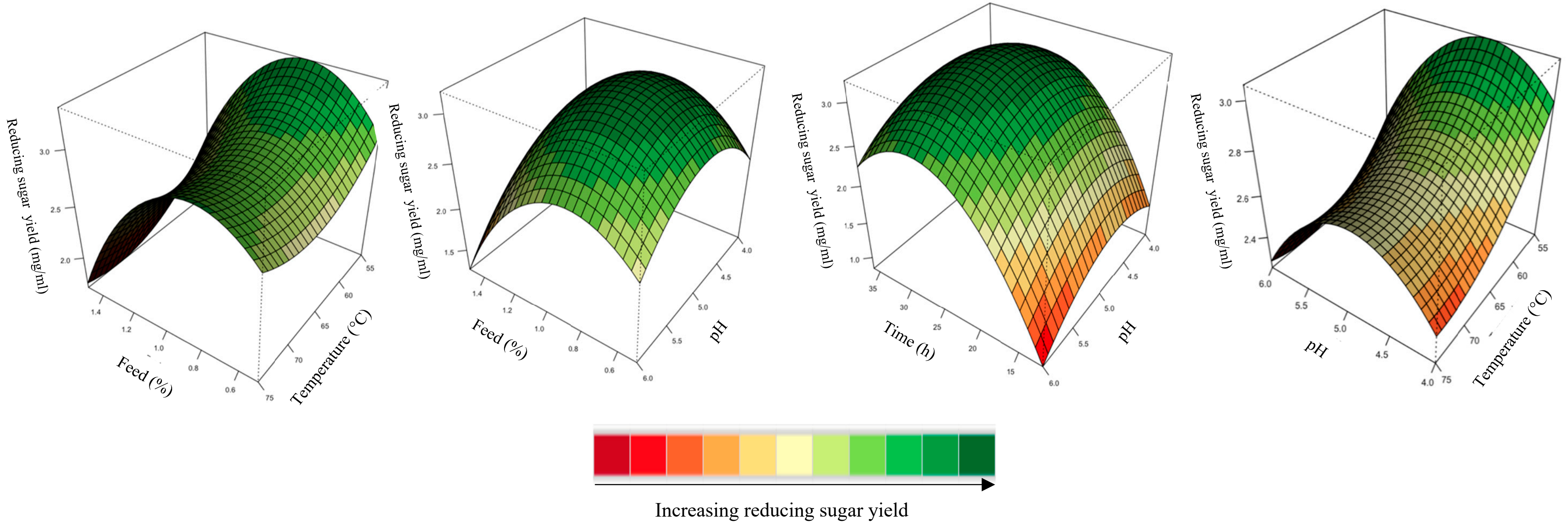

d

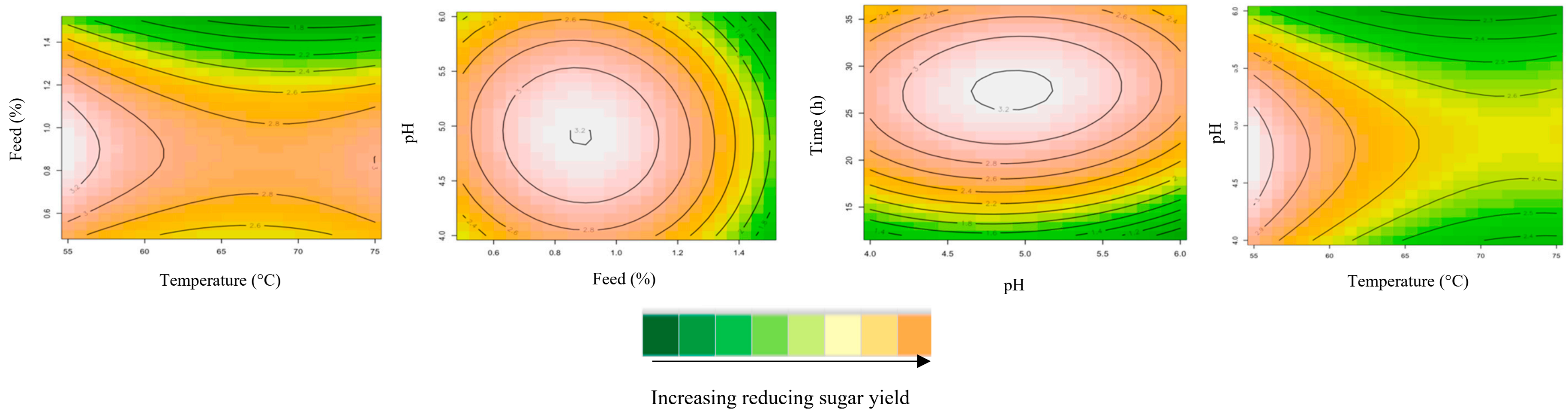

Figure S1: 3D- response surface plots (a and c) and contour plots (b and d) of the combined effects of variables on reducing sugar yield by crude *T. harzianum* xylanase of starter chicken feed.

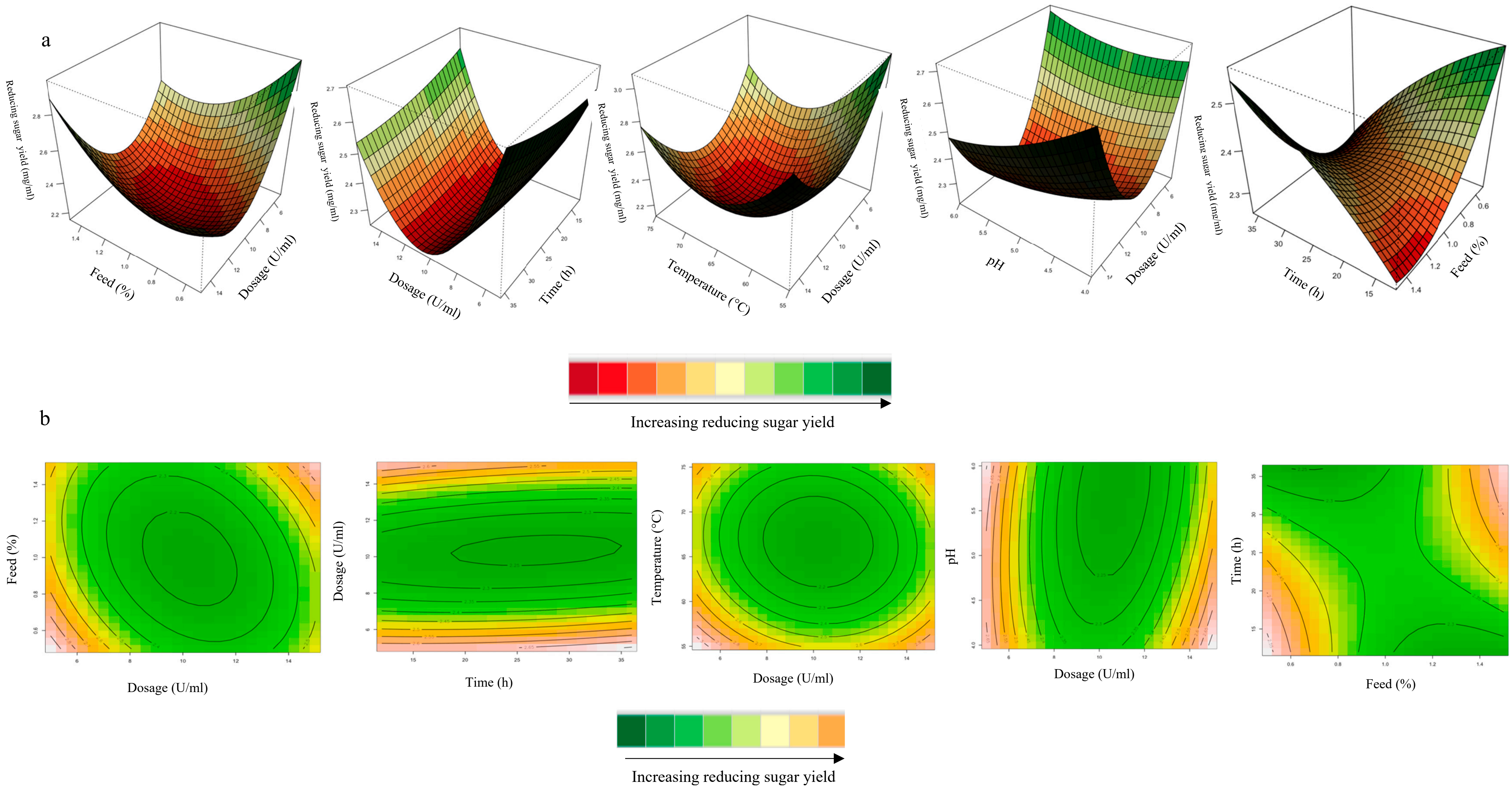
a
Reducing sugar yield (mg/ml)
Feed (%)
Dosage (U/ml)
Reducing sugar yield (mg/ml)
Dosage (U/ml)
Time (h)
Reducing sugar yield (mg/ml)
Temperature (°C)
Dosage (U/ml)
Reducing sugar yield (mg/ml)
pH
Dosage (U/ml)
Reducing sugar yield (mg/ml)
Time (h)
Feed (%)
Increasing reducing sugar yield
b
Feed (%)
Dosage (U/ml)
Dosage (U/ml)
Time (h)
Temperature (°C)
Dosage (U/ml)
pH
Dosage (U/ml)
Time (h)
Feed (%)
Increasing reducing sugar yield

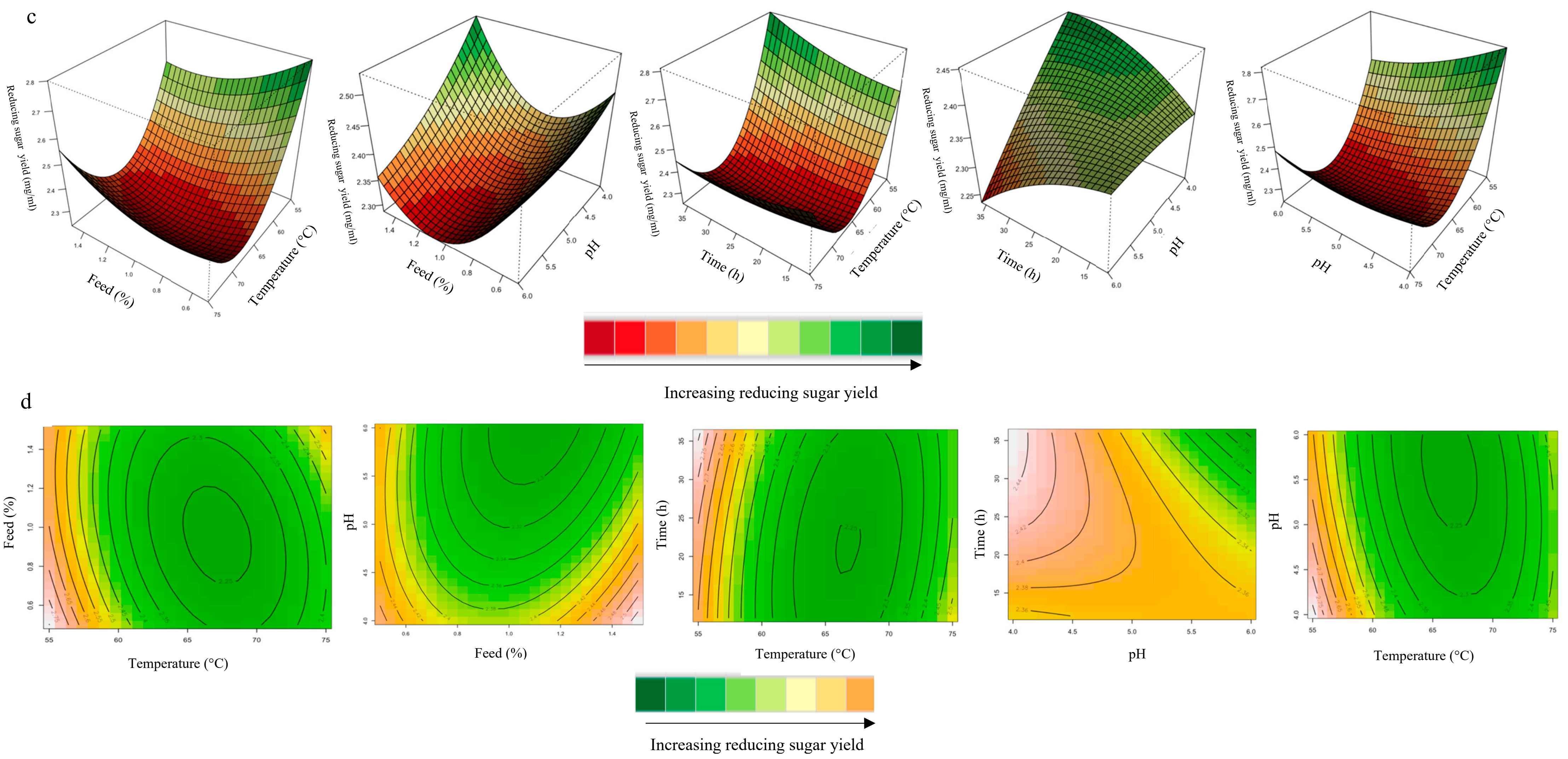

Figure S2: 3D- response surface plots (a and c) and contour plots (b and d) of the combined effects of variables on reducing sugar yield by crude *T. harzianum* xylanase of grower chicken feed.

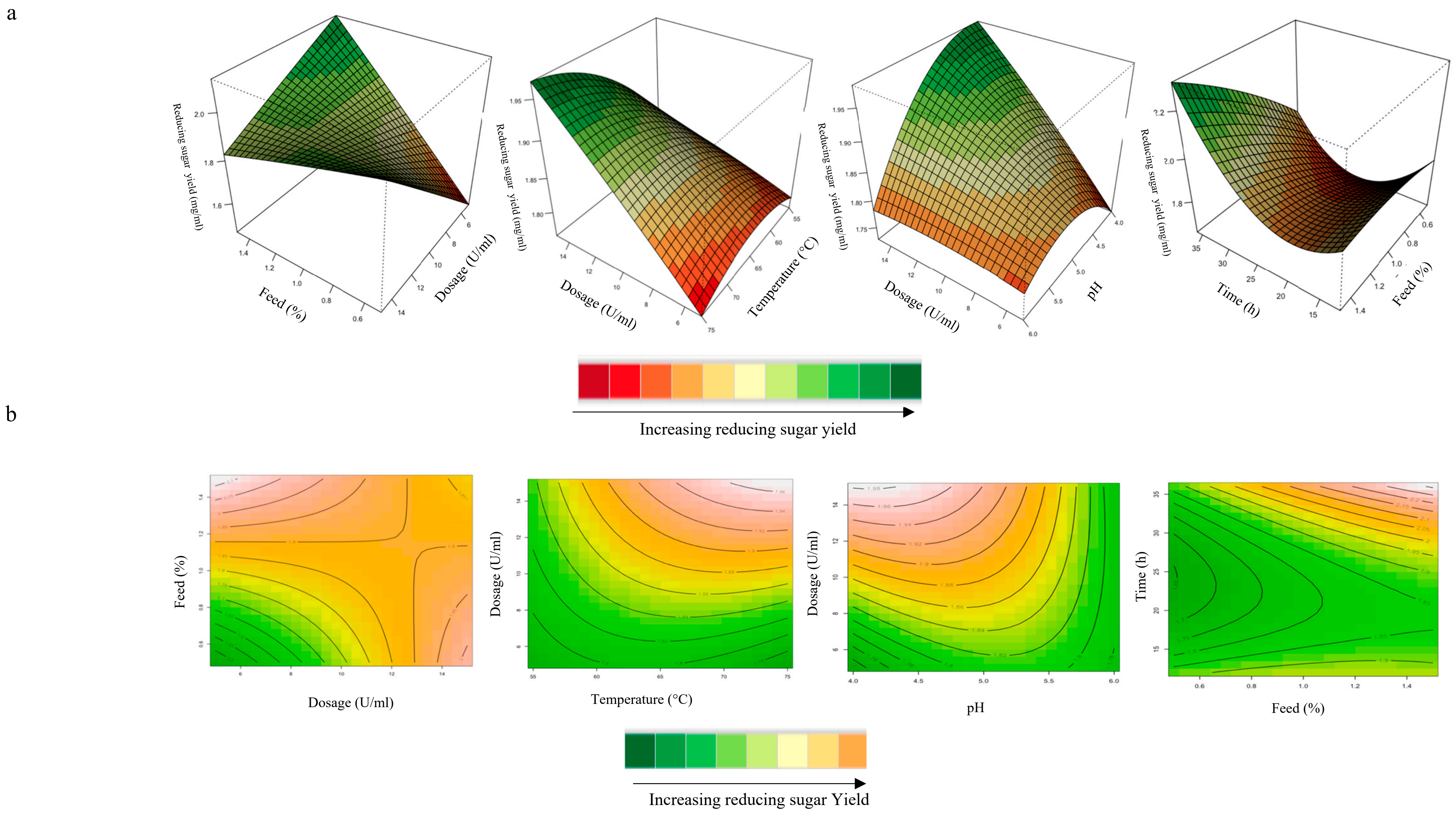
a
Reducing sugar yield (mg/ml)
Feed (%)
Dosage (U/ml)
Temperature (°C)
pH
Time (h)
Increasing reducing sugar yield
b
Feed (%)
Dosage (U/ml)
Temperature (°C)
pH
Time (h)
Increasing reducing sugar Yield

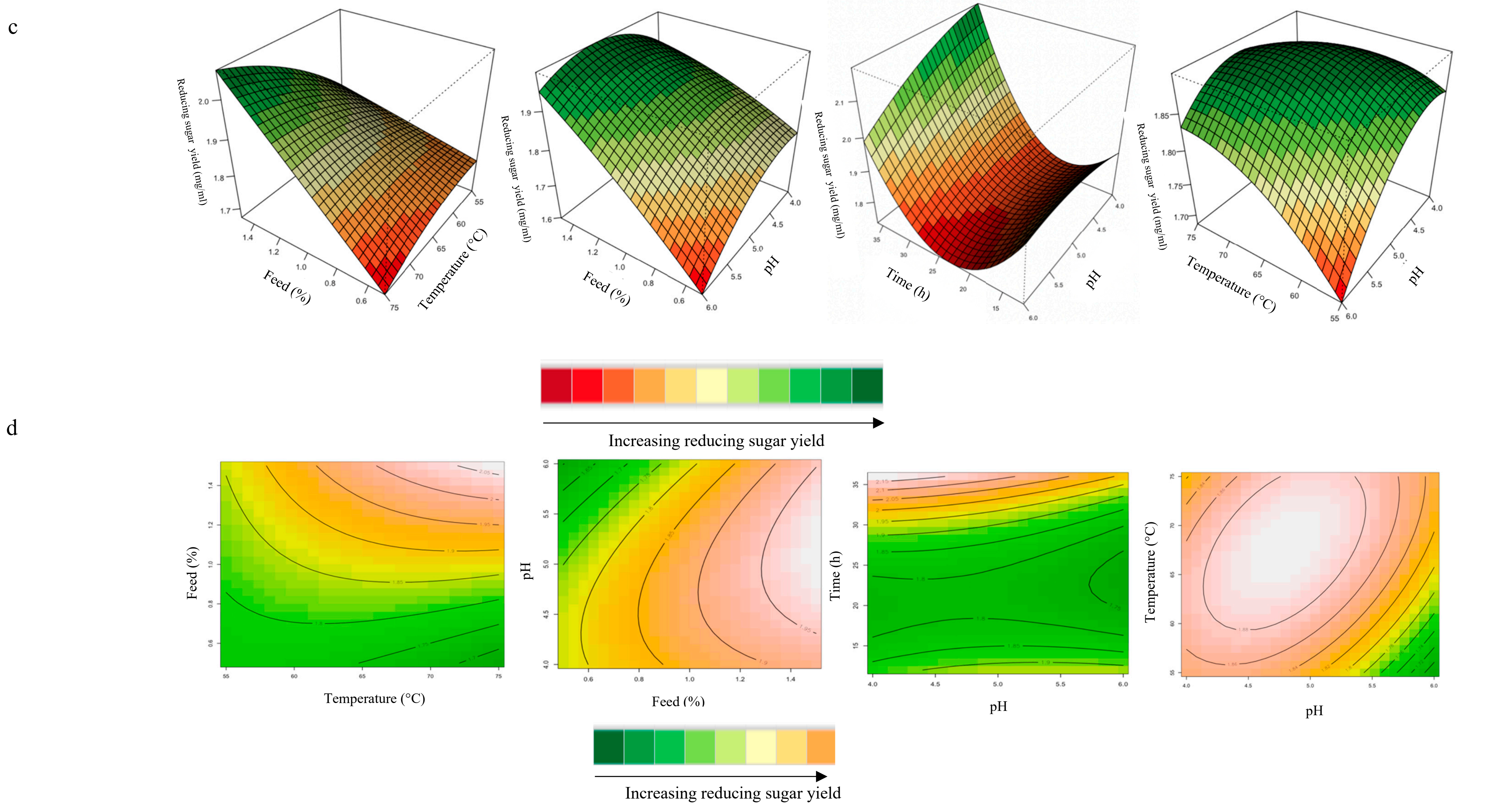

Figure S3: 3D- response surface plots (a and c) and contour plots (b and d) of the combined effects of variables on reducing sugar yield by purified *T. harzianum* xylanase of starter chicken feed.

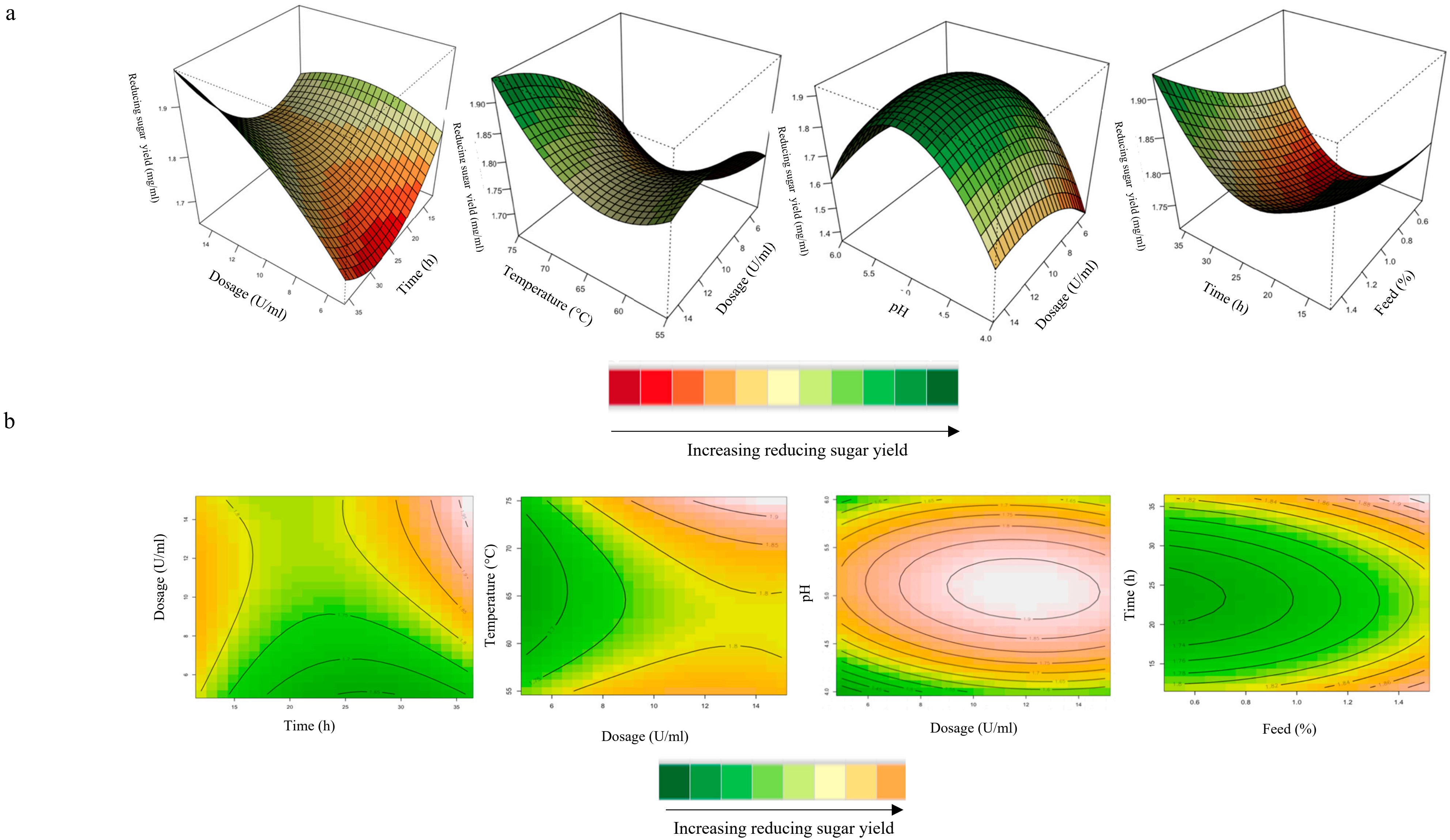
a
b
Reducing sugar yield (mg/ml)
Dosage (U/ml)
Time (h)
Temperature (°C)
pH
Feed (%)
Increasing reducing sugar yield
Increasing reducing sugar yield

c

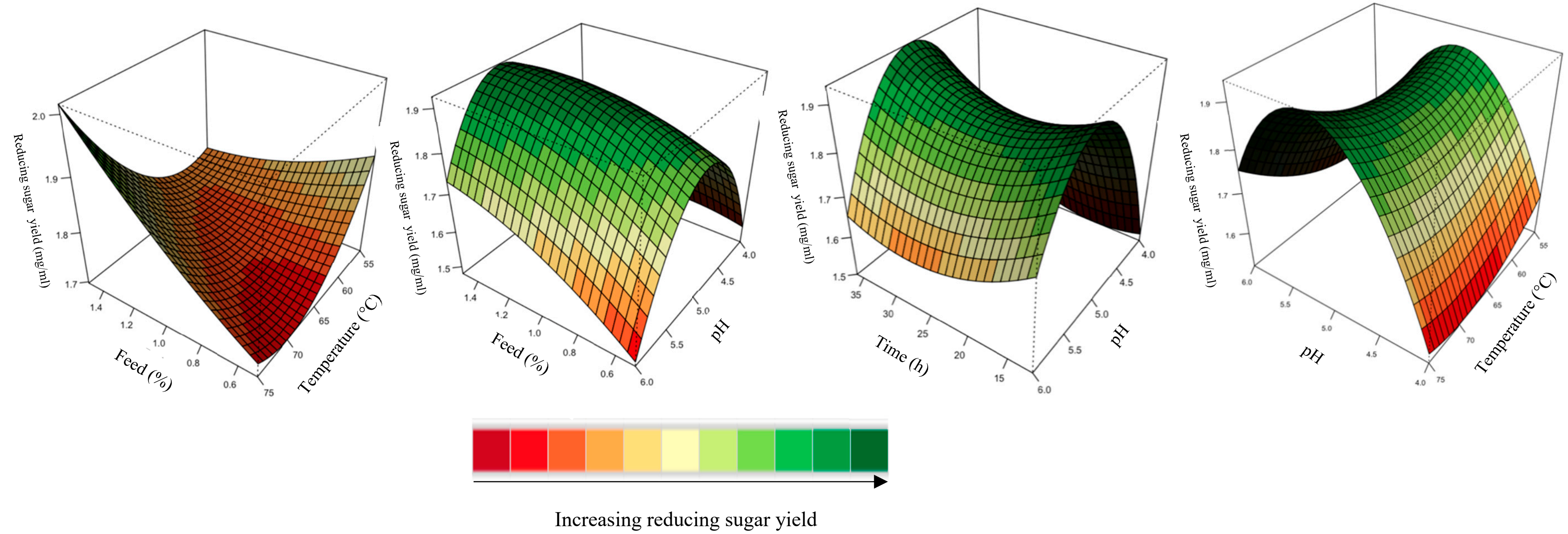

d

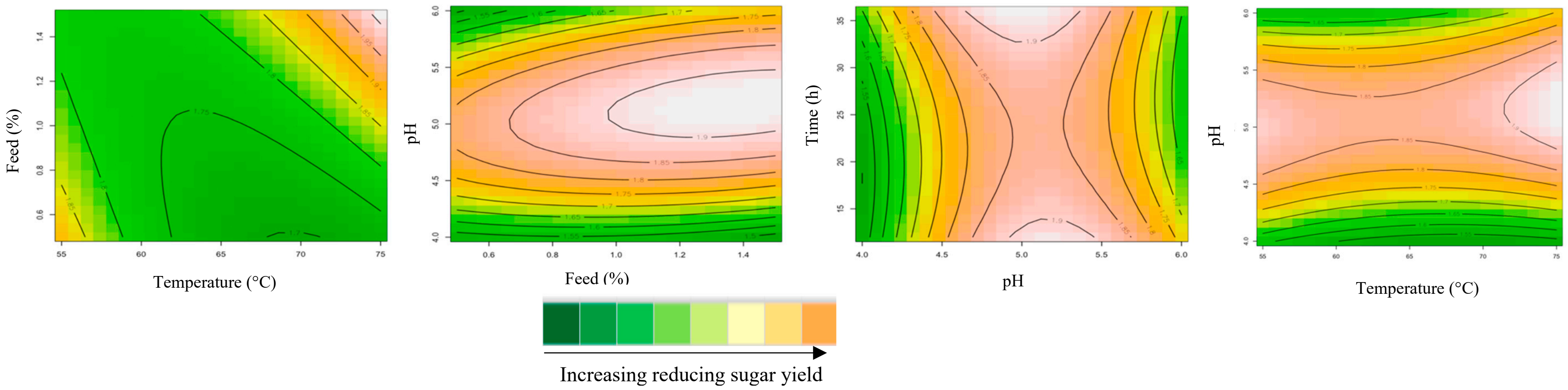

Figure S4: 3D- response surface plots (a and c) and contour plots (b and d) of the combined effects of variables on reducing sugar yield by purified *T. harzianum* xylanase of grower chicken feed.

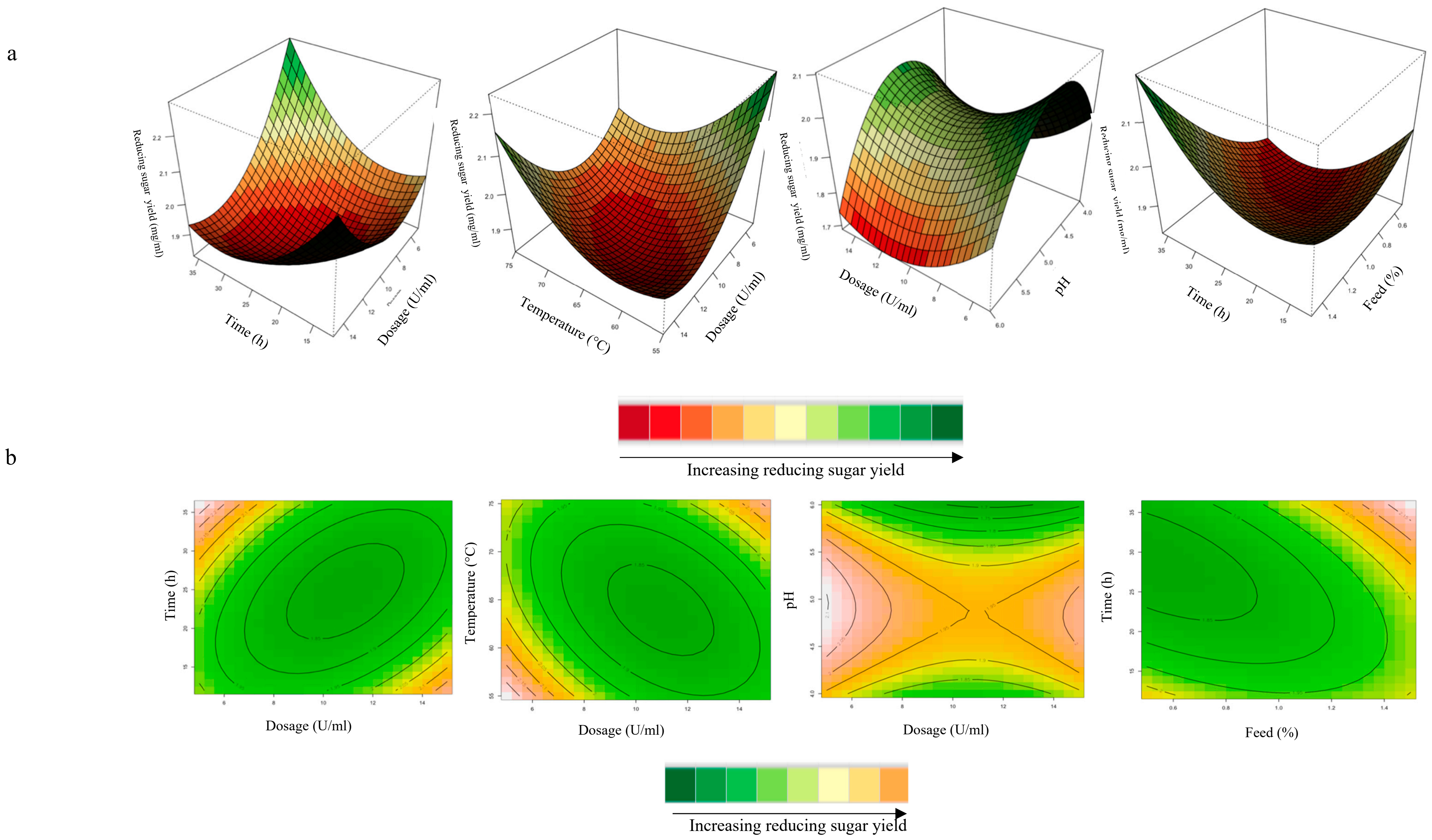
a
Reducing sugar yield (mg/ml)
Time (h)
Dosage (U/ml)
Temperature (°C)
pH
Feed (%)
Increasing reducing sugar yield
b
Time (h)
Dosage (U/ml)
Temperature (°C)
pH
Feed (%)
Increasing reducing sugar yield

c

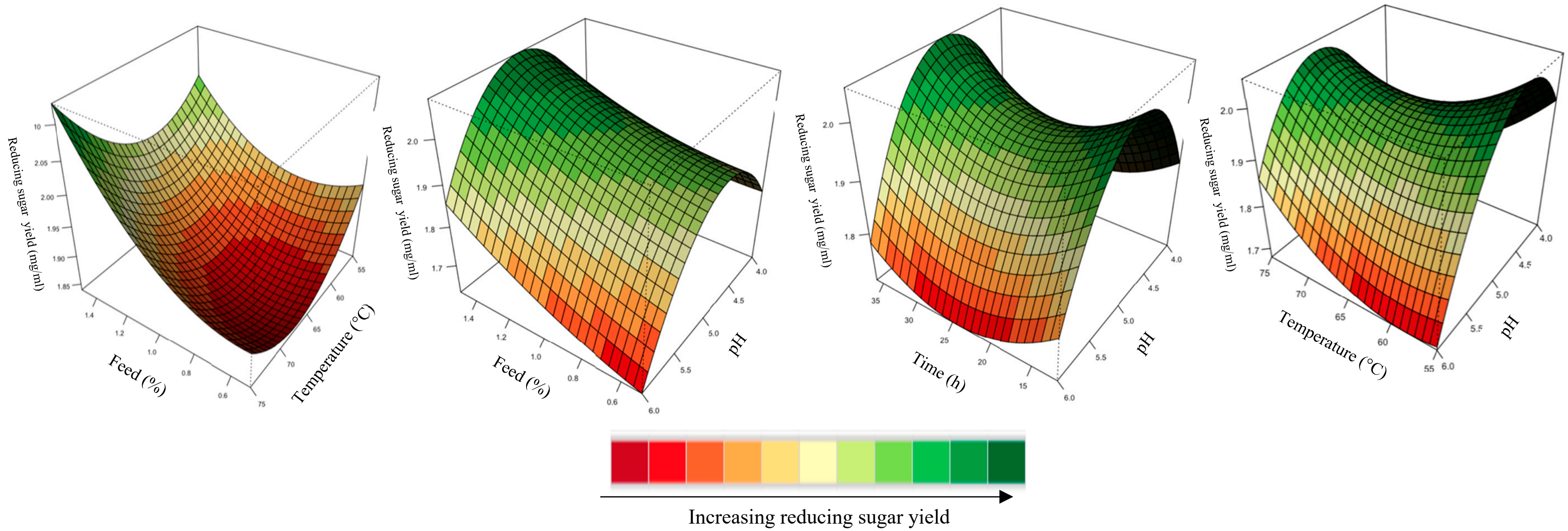

d

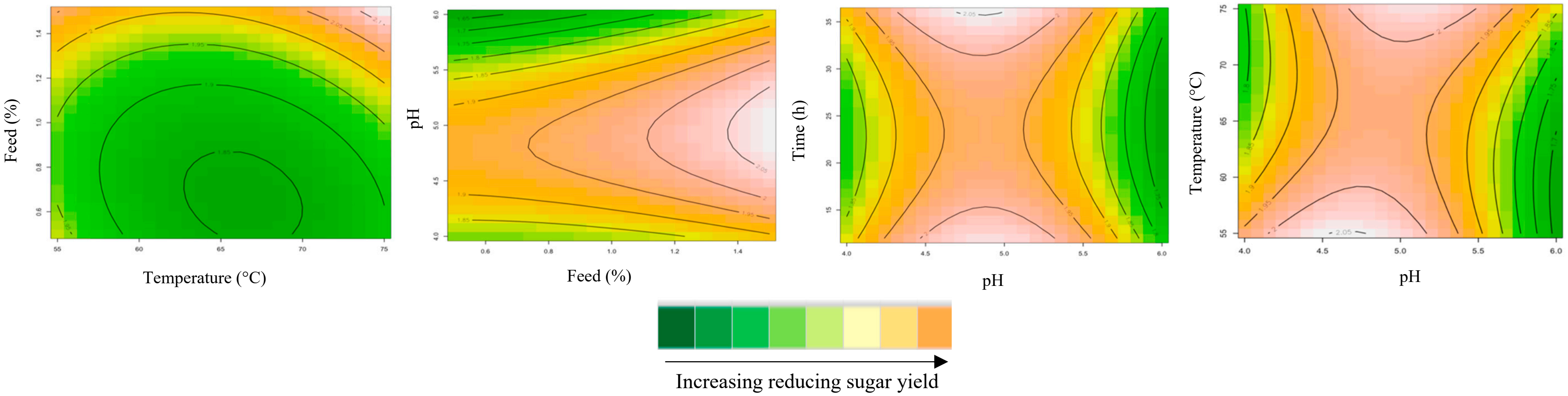

Figure S5: 3D- response surface plots (a and c) and contour plots (b and d) of the combined effects of variables on reducing sugar yield by purified XT6 of starter chicken feed.

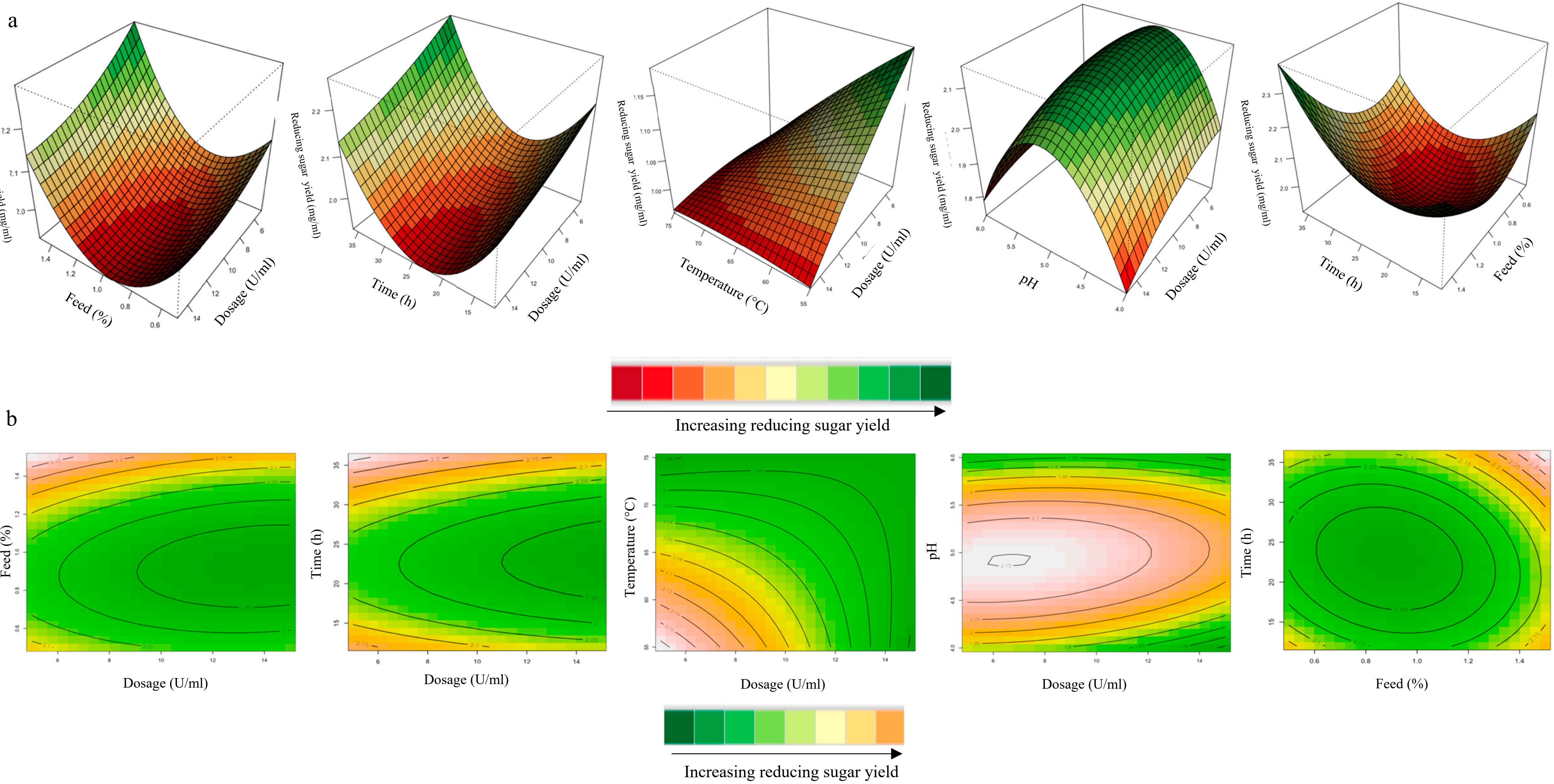
a
Reducing sugar yield (mg/ml)
Feed (%)
Dosage (U/ml)
Time (h)
Temperature (°C)
pH
Increasing reducing sugar yield
b
Feed (%)
Dosage (U/ml)
Time (h)
Dosage (U/ml)
Temperature (°C)
Dosage (U/ml)
pH
Dosage (U/ml)
Time (h)
Feed (%)
Increasing reducing sugar yield

c

d

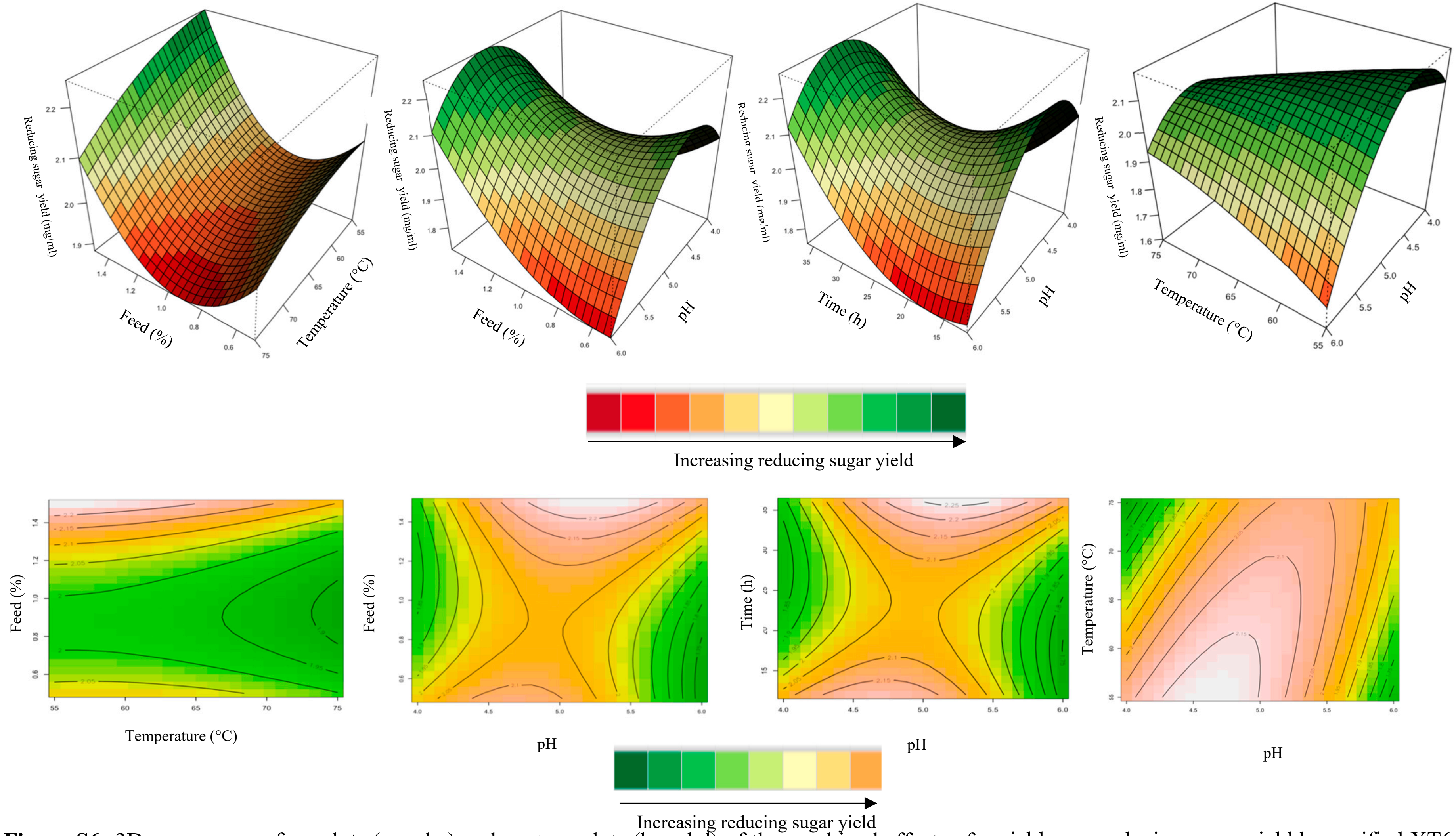

Figure S6: 3D- response surface plots (a and c) and contour plots (b and d) of the combined effects of variables on reducing sugar yield by purified XT6 of grower chicken feed.
